# Soy Protein-Cultured Mesenchymal Stem Cell-Secreted Extracellular Vesicles Target the Neurovascular Unit: Insights from a Zebrafish Brain Injury Model

**DOI:** 10.1021/acsbiomaterials.4c02304

**Published:** 2025-02-25

**Authors:** Tai-I Lin, Pei-Ying Hsieh, Hui-Jen Lin, Cheng-Kang Chiang, Jim Jinn-Chyuan Sheu, Wei-Tien Chang, Ian Liau, Hsin-Yun Hsu

**Affiliations:** † Department of Applied Chemistry and Institute of Molecular Science, National Yang-Ming Chiao-Tung University, Hsinchu 300093, Taiwan; ‡ Department of Chemistry, National Dong Hwa University, Hualien 974301, Taiwan; § Institute of Biomedical Sciences, National Sun Yat-Sen University, Kaohsiung 804201, Taiwan; ∥ National Taiwan University Hospital/National Taiwan University, Taipei 100233, Taiwan; ⊥ Center for Emergent Functional Matter Science, National Yang-Ming Chiao-Tung University, Hsinchu 300093, Taiwan

**Keywords:** extracellular vesicles, neurovascular unit, zebrafish model, soy protein isolates

## Abstract

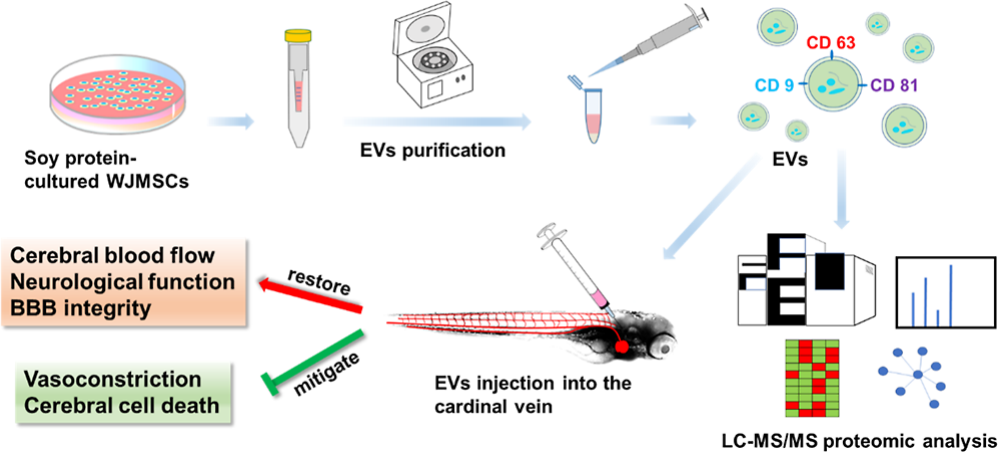

Cerebral vascular disorders often accompany hypoxia-induced brain injury. In this study, we develop a zebrafish model of hypoxia-induced cerebral vascular injury to replicate the associated phenotypic changes, including cerebrovascular damage, neuronal apoptosis, and neurological dysfunction. We then explored the therapeutic potential of extracellular vesicles derived from Wharton’s jelly-derived mesenchymal stem cells (WJ-MSCs) cultured on soy protein-coated surfaces. These vesicles demonstrated superior recovery efficacy, especially in restoring the blood–brain barrier integrity and improving neurological function. Our findings suggest that these potent therapeutic extracellular vesicles, easily produced from WJ-MSCs cultured in the presence of soy proteins, may mitigate hypoxia-induced brain injury by decreasing the severity of vascular disorder caused by oxidative stress. Protein–protein interactome analysis further suggests that multiple signaling pathways are likely involved in restoring normal neurovascular unit function.

## 1 Introduction

Hypoxia-induced brain injury involves a continuous neurotoxic process instead of an event. Energy failure, cerebral edema, and cerebral microvascular damage occur within a few hours of hypoxia, causing the loss of the membrane integrity of neurons. After reoxygenation and reperfusion, sequential reactions such as inflammation, oxidative stress, and excitotoxicity are activated, which subsequently lead to severe microvascular damage. This process can last from days to weeks, ultimately resulting in cell death and severe neuronal damage.^1,2^ It has been known that neurovascular interactions are important in brain hemodynamics and maintenance of the blood–brain barrier (BBB).^3,4^ The structure and integrity of blood vessels play critical roles in maintaining an adequate cerebral blood supply and normal brain function.^5^ Previous studies on models of perinatal injury in term infants showed that hypoxic–ischemic brain injury involved damage of the inner vascular endothelial cells and the loss of BBB integrity.^6^ An increased BBB permeability (an increased ratio of albumin in the plasma and the cerebrospinal fluid) in neonates with perinatal asphyxia was observed.^7^ Alternatively, protecting the neurovascular unit (NVU), which is the structural and functional multicellular modules comprised of endothelial cells, pericytes, perivascular astrocytes, the extracellular matrix, and neurons, has been found to reduce neuronal apoptosis and brain damage in a rat model of neonatal hypoxic–ischemic brain injury.^8^ In light of the importance of the NVU in brain function, NVU might serve as a feasible therapeutic target for hypoxia-induced brain injury, which is worth exploring.

As a result, experimental animal models are important for exploring underlying diseases and developing therapeutics. The Rice–Vannucci model has been used to study neonatal hypoxic–ischemic brain injury.^9^ In this model, a 7 day postnatal rat was used, and the unilateral common artery was permanently ligated. Using this model, many studies have reported that cerebral hypoxia caused a blood flow decrease, microvascular damage, BBB impairment, neuroinflammation, and neuronal apoptosis.^2,8,10^ Unfortunately, the infarct severity after surgery in the model has a high variation,^11,12^ making the comparison of results among experimenters difficult. Zebrafish (*Danio rerio*) thus becomes an attractive alternative model organism because of its high fertility, reduced time and cost, possibility of noninvasive longitudinal observation of the change of the vascular structure and function, neural damage through optical imaging, thereby offering remarkable convenience for phenotype-based therapeutic assessment.^13^ Adult zebrafish (3 months) have been employed to develop the model for hypoxic–ischemic brain injury.^14^ Hypoxic injury caused erratic movements, including circling and rotating in adult zebrafish, and triphenyl tetrazolium chloride staining showed brain damage. A recent study further showed that zebrafish larvae subject to hypoxia and reoxygenation treatment caused neuronal dysfunction and glial activation.^15^ Nevertheless, none of these zebrafish studies have systematically observed that the vascular or BBB changes occurred during cerebral hypoxia and reoxygenation.

Apart from the pursuit of robust experimental animal models, there is also an urgent need for new treatments of hypoxia-induced brain injury. Therapeutic hypothermia^16^ remains the standard of care in clinics; however, its efficacy is limited, especially in moderate-to-severe patients.^17^ Although alternative strategies such as stem-cell transplant^18^ and erythropoietin therapy^19^ have emerged, their efficacy was also unsatisfactory. Recently, cell-derived extracellular vesicles (EVs) such as exosomes have been found to mediate cell-to-cell communication. The richness of its content (e.g., microRNAs, proteins, and metabolites) facilitated intercellular signaling within the NVU, revealing the restorative therapeutic potential of exosomes harvested from multipotent mesenchymal stem cells to treat ischemic stroke.^20^ The exosomes could cross the blood–brain and blood–cerebrospinal fluid barriers to inhibit neuroinflammation and modulate brain injury,^21^ and thus they have been proposed as a promising therapeutic shuttle of natural nanoparticles.^22^ Several studies indicated while exosomes could modulate the microenvironment, the micromilieu could also affect the exosomal secretion and its composition.^23,24^ The secreted exosomes could be regulated even by the nanomorphology of culture substrates, memorizing the cellular delivery information on biomaterials.^25^ To upscale the bioactivity and the yield, exosomes also had been engineered via means of chemical and biological modulations^26^ or mechanical stimulation by fluid shear flow.^27^ Despite significant efforts in the field, our understanding in developing therapeutic exosomes remains scarce.^28^

Herein, we established a zebrafish model that mimics hypoxic–ischemic brain injury and to verify whether the hypoxic–ischemic brain injury zebrafish exhibits a resembled phenotype change, such as cerebrovascular damage, neuronal apoptosis, and neurological dysfunction, similar to that found in infants or experimental mammalian models. In parallel, using this model, we investigated the therapeutic potential of the exosomes collected from soy protein-cultured Wharton’s jelly mesenchymal stem cells (WJ-MSCs) to ameliorate hypoxia-induced neurovascular damage. The plant-based proteins such as soy proteins^29^ have revealed multiple bioactivities, including the scavenging of free radicals and neuroprotection from stroke.^30^ As exosome secretion has been found to be able to be altered by its surroundings,^24^ we wondered whether the MSC-derived exosomes could be manipulated by facile culture of cells on a soy protein-coated surface to improve its potency. Distinct from existing studies, in which either enrichment of exosomes with specific microRNAs^22^ or ligand functionalization and additional drug doping were required to enhance the therapeutic efficacy,^31^ we found that significant improvement in observable phenotypes of hypoxic–ischemic brain injury zebrafish could be achieved by simply applying such soy protein-cultured, WJ-MSC-derived exosomes. Finally, proteomics analysis was performed to explore the likely regulatory pathways.

## 2 Experimental Section

### 2.1 Preparation of the Soy Protein-Coated Culture Plate

Soy protein isolate (0.5 g) was dissolved in 0.02 M Na_2_CO_3_ (10 mL with 3% glycerol) and incubated at 80 °C for 60 min with the vortex (vortex twice at 80 rpm every 20 min). The heated protein mixture was then centrifuged at 1500 rpm for 5 min and the supernatant was collected. The supernatant (2 mL) was then added to coat the 10 cm cell culture dish and air-dried overnight in the biosafety cabinet.

### 2.2 Collection and Characterization of Exosomes

WJ-MSCs (1 × 10^6^) were cultured, respectively, on the soy protein-coated and uncoated cell culture dish using WJ-MSC cell culture medium (MEM supplemented with 10% FBS and 1% penicillin/streptomycin) for 24 h, and the cultured medium was removed and replaced with 10 mL of MEM (with 0.5% BSA) for another 24 h incubation. The supernatant was collected and centrifuged at 2000*g* for 10 min to remove residual cells and cell debris if present, followed by 40× (40 to 1 mL) concentration using a Vivaspin Turbo 15 (100 K MWCO) concentrator centrifugal tube by centrifugation at 4 °C and 4000*g* for 10 min which contains EVs. To obtain exosomes, the sample was again centrifuged at 14,000*g* for 1 h, and the supernatant was collected and added to ExoQuick-TC exosome precipitation solution (Cat. no. EXOTC10A-1, SBI) and allowed to stand overnight at 4 °C. Exosome precipitation was then obtained by centrifugation at 1500*g* for 30 min and washed twice with 50 μL PBS. The isolated exosomes were stored at 4 °C and used within 3 days. The purified exosomes were labeled with the lipophilic tracer DiD (Thermo Fisher) and counted in a high-resolution fluorescence imaging flow analyzer (ImageStreamX Mark II, Amnis; NSTC Basic Research Core Facility, NYCU). Exosomes (8 × 10^7^/mL) were characterized by the presence of CD9, CD63, and CD81 markers using respective antibodies (Proteintech Group, Inc.) with 1:100 dilution by PBS, followed by 20 min incubation at room temperature in the dark and analyzed by ImageStreamX Mark II. To obtain exosomal proteins for Western blot and for LC–MS/MS analysis, purified exosome precipitates were treated with 50 μL cell lysis buffer CelLytic M (Sigma) containing phosphatase inhibitor cocktails and incubated 10 min on ice, followed by centrifugation at 15000 rpm, 4 °C for 10 min, and the supernatant was collected for protein quantification.

### 2.3 Administration of Exosomes

To explore the therapeutic effects of exosomes against hypoxia-induced brain injury, we randomly assigned zebrafish larvae to three groups: Exo_Soy, Exo_Control, and Vehicle. The Exo_Soy group consisted of larvae subjected to a hypoxic insult, followed by an injection of exosomes collected from soy protein-cultured WJ-MSCs. The Exo_Control group included larvae subjected to the same hypoxic insult and injected with WJ-MSC-secreted exosomes collected from uncoated culture dishes. The Vehicle group comprised larvae subjected to the hypoxic insult and injected with the medium alone. Immediately after the designated hypoxia episode, exosome suspensions (i.e., Exo_Soy and Exo_Control) containing 4 × 10^4^ particles in 0.5 μL were administered via injection into the larvae’s cardinal vein. The Vehicle group underwent the same injection procedure but received 0.5 μL of an exosome-free medium. This ensured that any observed effects could be attributed to the exosomes themselves rather than the injection process or the medium used.

### 2.4 Statistical Analysis

All statistical analyses were performed using SPSS software. For experiments involving repeated measurements of the same group at different time points—excluding those assessing BBB impairment—a paired *t*-test was used to compare means between two time points. For BBB impairment assessments, which involved comparisons between two independent groups, an unpaired two-sample *t*-test was employed. Statistical significance was determined at three levels: *p < 0.05, **p < 0.01, and ***p < 0.001; NS indicates no significant difference (*p* ≥ 0.05).

## 3 Results

### 3.1 Zebrafish Model of Hypoxia-Induced Brain Injury

#### 3.1.1 Dependence of Larval Survival on the Duration of Hypoxic Insult

To establish the disease model, we first assessed the survival of zebrafish larvae (6 days postfertilization, dpf) following various durations of hypoxic exposure. Survival was monitored at specific time points posthypoxia, as detailed in the Supporting Information (Figure S1). Survival curves over the 48 h posthypoxia period demonstrate a clear relationship between hypoxic duration and survival outcomes. At 48 h postinsult, survival rates were 95 ± 5% following 5 min of hypoxia, 86 ± 8% for 10 min, 47 ± 11% for 15 min, and 13 ± 18% for 20 min. These data demonstrate a clear inverse relationship between the duration of hypoxic exposure and survival with longer exposures leading to progressively lower survival rates. Based on these findings, we selected the 15 min hypoxic insult for subsequent experiments, as it produced approximately 50% survival at 48 h. This duration provides an optimal balance between injury severity and survivability, making it a suitable model for studying hypoxic–ischemic brain injury and assessing potential therapeutic interventions.

#### 3.1.2 Cerebral Cell Viability and Neurological Dysfunction Following a 15 min Hypoxic Insult

**Figure 1:**
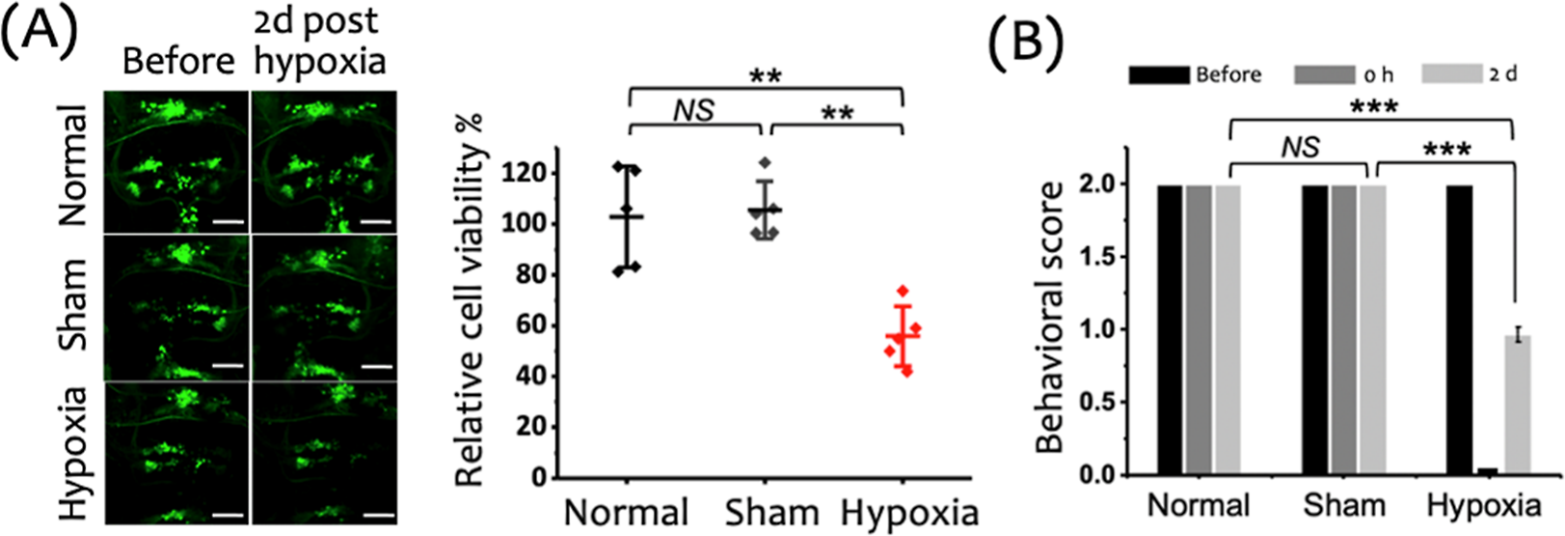
Evaluation of cerebral cell viability and neurological deficits in zebrafish larvae following a 15 min hypoxic insult. (A) Left: Representative fluorescence images of cranial motor neurons (green: GFP) in zebrafish larvae, captured before and 2 days after the hypoxic insult. Scale bar: 50 μm. Right: Quantification of relative cerebral cell viability reveals a significant decrease in the Hypoxia group compared to the Normal and Sham groups. Data are shown as mean ± SD with individual data points (*n* = 5 larvae per group). (B) Behavioral assessment of neurological function in zebrafish larvae conducted before the hypoxic insult (before), immediately after (0 h), and 2 days posthypoxia (2 d). Behavioral scores significantly declined in the Hypoxia group compared to both Normal and Sham groups at 2 days postinsult. Data are presented as mean ± SD from three independent experiments (*n* = 30 larvae per experiment). ***p < 0.001, **p < 0.01, *p < 0.05, and NS: not significant.

To assess the impact of a 15 min hypoxic insult on cerebral cell viability and neurological function, zebrafish larvae were divided into three groups: Normal, Sham, and Hypoxia. Representative fluorescence images of the cranial region of zebrafish larvae, taken before and 2 days after the hypoxic insult, are shown in Figure 1A (left panel). In the Hypoxia group, dimmer fluorescence signals were observed compared to those of the Normal and Sham groups, indicating a reduction in cerebral cell viability. Quantitative analysis, detailed in the Supporting Information (Figure S2), revealed relative cerebral cell viabilities of 103 ± 20% in the Normal group, 106 ± 11% in the Sham group, and 56 ± 12% in the Hypoxia group (Figure 1A, right panel). Statistical analysis confirmed that cerebral cell viability in the Hypoxia group was significantly lower than in both Normal group (*p* < 0.01) and the Sham group (*p* < 0.01). No significant difference was observed between the Normal and Sham groups (*p* > 0.05).

Neurological function of zebrafish larvae was assessed through behavioral testing, as described in the Supporting Information, with results summarized in Figure 1B. Prior to the hypoxic insult, all larvae exhibited normal neurological behavior (Behavioral score = 2). Immediately following the 15 min hypoxic exposure, larvae in the Hypoxia group became motionless and unresponsive to external stimuli (Score = 0), indicating severe neurological impairment. By 2 days posthypoxia, many larvae in the Hypoxia group exhibited persistent deficits, with a significantly reduced behavioral score (0.97 ± 0.05), compared to the Normal and Sham groups (*p* < 0.001). In contrast, larvae in the Normal and Sham groups maintained normal behavioral function (Score = 2) throughout the experiment, with no significant differences between them (*p* > 0.05).

Together, these findings demonstrate that a 15 min hypoxic insult significantly reduces cerebral cell viability and induces severe, lasting neurological deficits in zebrafish larvae, as confirmed by both cellular viability and behavioral assessments.

#### 3.1.3 Impairment of the NVU Following a 15 min Hypoxic Insult

**Figure 2:**
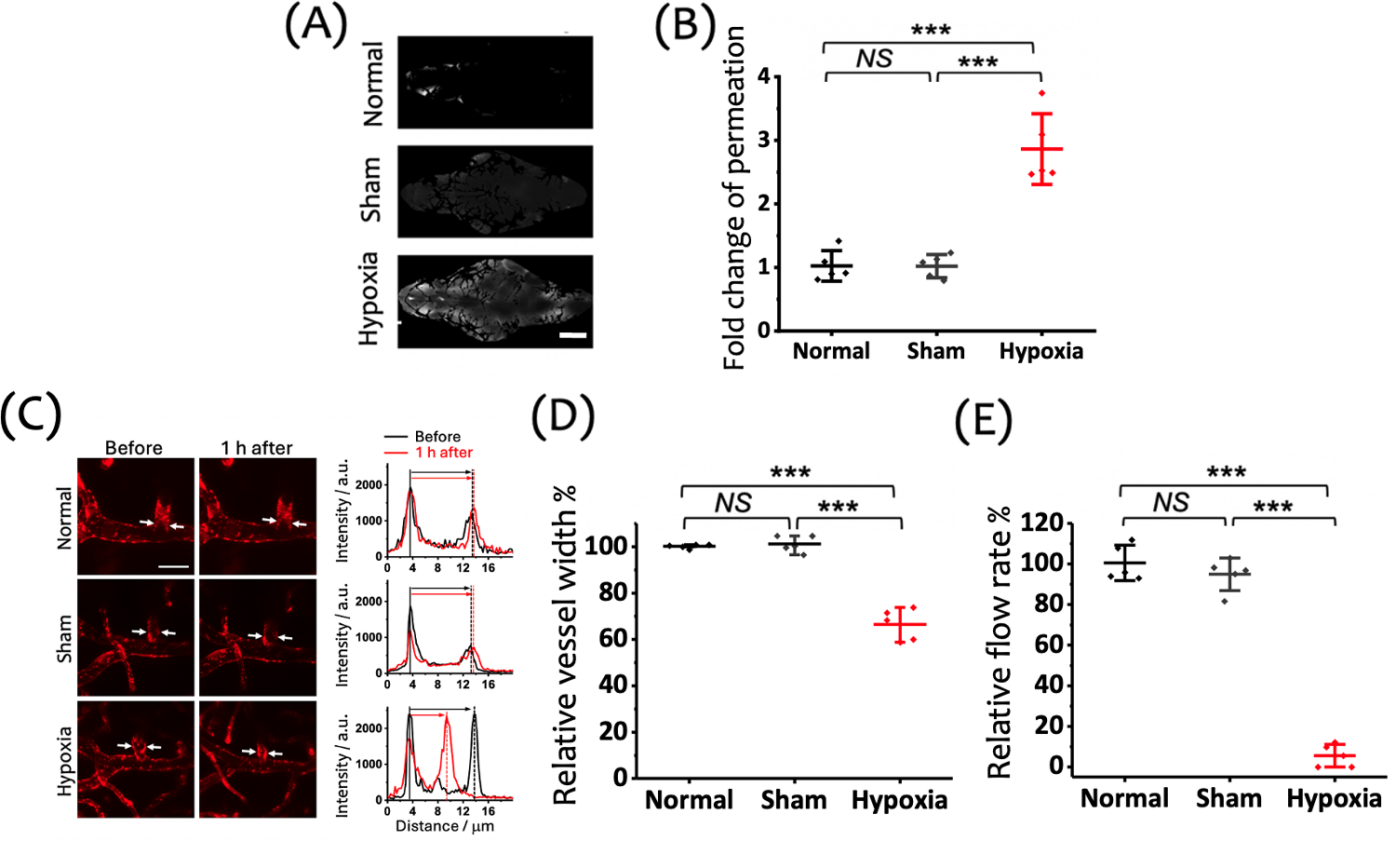
Effects of a 15 min hypoxic insult on BBB integrity, vasculature structure, and cerebral blood flow in zebrafish larvae, assessed 1 h postinsult. (A) Representative images of the cranial region showing the extravascular accumulation of fluorescent tracers (gray: RITC-dextran, MW: 10 k), 1 h posthypoxia following vascular injection, indicating BBB disruption in the Hypoxia group. Scale bar: 100 μm. (B) Fold change in tracer permeation, highlighting a significant increase in BBB permeability in the Hypoxia group compared to the Normal and Sham groups. (C) Left: Representative images of cerebral vasculature (red: mCherry) in zebrafish larvae before and 1 h after the 15 min hypoxic insult. White arrows indicate the proximal region of the first branch of the central artery (CtA1st), where vessel width measurements were taken. Scale bar: 20 μm. Right: Cross-sectional fluorescence intensity profiles showing vessel width changes pre- and posthypoxia. (D) Quantification of the relative vessel width in the CtA1st across the Normal, Sham, and Hypoxia groups, showing a significant reduction in the Hypoxia group. (E) Relative flow rate in the CtA1st, demonstrating a significant reduction in the Hypoxia group compared to the Normal and Sham groups. Data are shown as mean ± SD with individual data points (*n* = 5 larvae per group) in panels (B), (D), and (E).

To investigate the impact of a 15 min hypoxic insult on the NVU, we evaluated BBB integrity, vessel width of the first branch of the central artery (CtA1st), and cerebral blood flow, as detailed in the Supporting Information (Figures S3–S5, respectively). Figure 2A shows representative images of extravascular accumulation of fluorescent tracers in the cranial region of zebrafish larvae, taken 1 h after hypoxia. These images clearly demonstrate a marked disruption of the BBB in the Hypoxia group, evidenced by a significant increase in tracer permeation beyond the vasculature. Quantification of this permeability is presented in Figure 2B, with fold changes of 1 ± 0.2, 1 ± 0.2, and 2.9 ± 0.6 for the Normal, Sham, and Hypoxic groups, respectively. Statistical analysis revealed a substantial increase in BBB permeability in the Hypoxia group compared to the Normal (*p* < 0.001) and Sham groups (*p* < 0.001), while no significant difference was observed between the Normal and Sham groups (*p* > 0.05). These results indicate that even a brief hypoxic event significantly impairs BBB integrity.

Structural changes in the cerebral vasculature were assessed by comparing the vessel width before and after the hypoxic insult. Figure 2C (left panel) displays representative images of the cerebral vasculature, showing pronounced vasoconstriction in the Hypoxia group, with a visibly reduced vessel width 1 h postinsult. In contrast, the Normal and Sham groups exhibited no appreciable changes in the vessel width over time. Cross-sectional fluorescence intensity profiles (Figure 2C, right panel) further validate these observations, highlighting the reduction in the vessel width in the Hypoxia group. Quantitative analysis of the relative vessel width is shown in Figure 2D, where the widths were measured as 100 ± 1%, 101 ± 4%, and 67 ± 7% for the Normal, Sham, and Hypoxic groups, respectively. Statistical comparisons indicated a significant decrease in the vessel width in the Hypoxia group compared to both Normal (*p* < 0.001) and Sham groups (*p* < 0.001), with no significant difference between the Normal and Sham groups (*p* > 0.05).

Finally, we evaluated cerebral blood flow following hypoxia. As shown in Figure 2E, relative flow rates were measured at 100 ± 9%, 95 ± 8%, and 6 ± 6% for the Normal, Sham, and Hypoxic groups, respectively. Statistical analysis confirmed a significant reduction in the blood flow in the Hypoxia group compared to the Normal (*p* < 0.001) and Sham groups (*p* < 0.001), while no significant difference was observed between the Normal and Sham groups (*p* > 0.05). The marked reduction in the cerebral blood flow observed in the Hypoxia group is likely influenced by the vasoconstriction indicated by the vessel width measurements. However, since the decrease in the vessel width is less pronounced than the reduction in the flow rate, it suggests that additional factors may also contribute to the significant decline in perfusion.

### 3.2 Evaluation of the Therapeutic Efficacy of EVs on Hypoxia-Induced Brain Injury

**Figure 3:**
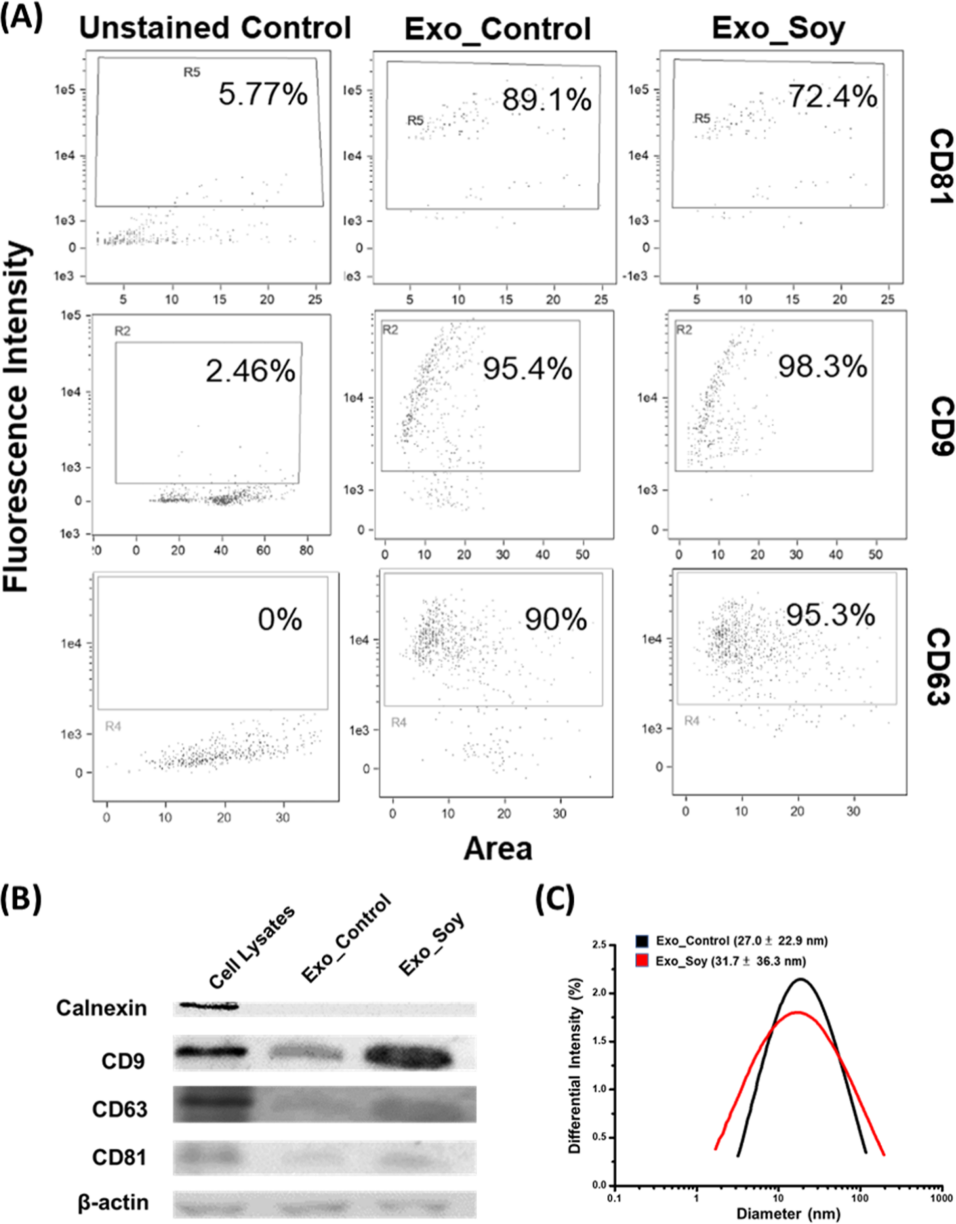
Characterization of exosomes. WJ-MSC-secreted exosomes collected from soy protein-coated (Exo_Soy) and uncoated (Exo_Control) culture dishes were, respectively, characterized for the surface markers CD81, CD9, and CD63 by (A) flow cytometric analyzer (ImageStreamX Mark II, Amnis) and (B) Western blot (Calnexin served as the negative marker of exosomes, and β-actin is the loading control). (C) DLS analysis was performed to characterize the respective diameters of these exosomes.

Plant-based proteins, such as soy proteins, have demonstrated various bioactivities,^32^ including free radical scavenging and neuroprotection in stroke. Based on previous evidence that exosome secretion can be influenced by external factors, we hypothesized that culturing MSCs in the presence of soy proteins could modulate the therapeutic efficacy of MSC-derived exosomes. The WJ-MSCs were cultured on soy protein-coated plates rather than directly cultured in the soy protein-containing medium due to the colloidal properties of soy proteins, which hindered subsequent exosome purification. To characterize the obtained exosomes, the flow cytometric imaging analysis^33^ was applied (Figures 3A and S6) and Western blotting (Figure 3B) further confirmed the presence of characteristic exosomal surface markers (CD9, CD63, and CD81) in exosomes derived from WJ-MSCs cultured on both soy protein-coated (Exo_Soy) and uncoated (Exo_Control) culture dishes. Calnexin serves as a negative control and was absent in both Exo_Control and Exo_Soy, indicating the purity of the prepared exosome samples. Notably, the size distribution of Exo_Soy was slightly broader compared to that of Exo_Control (Figure 3C). Toxicity assessment in zebrafish larvae showed an approximately 90% survival rate for Exo_Control and a full 100% survival for Exo_Soy (Figure S7), suggesting the improved biocompatibility of exosomes derived from WJ-MSCs cultured on soy protein-coated surfaces.

#### 3.2.1 Exosomes Improve Survival and Mitigate Neurological Dysfunction and Cerebral Cell Death Following Hypoxic Insult

**Figure 4:**
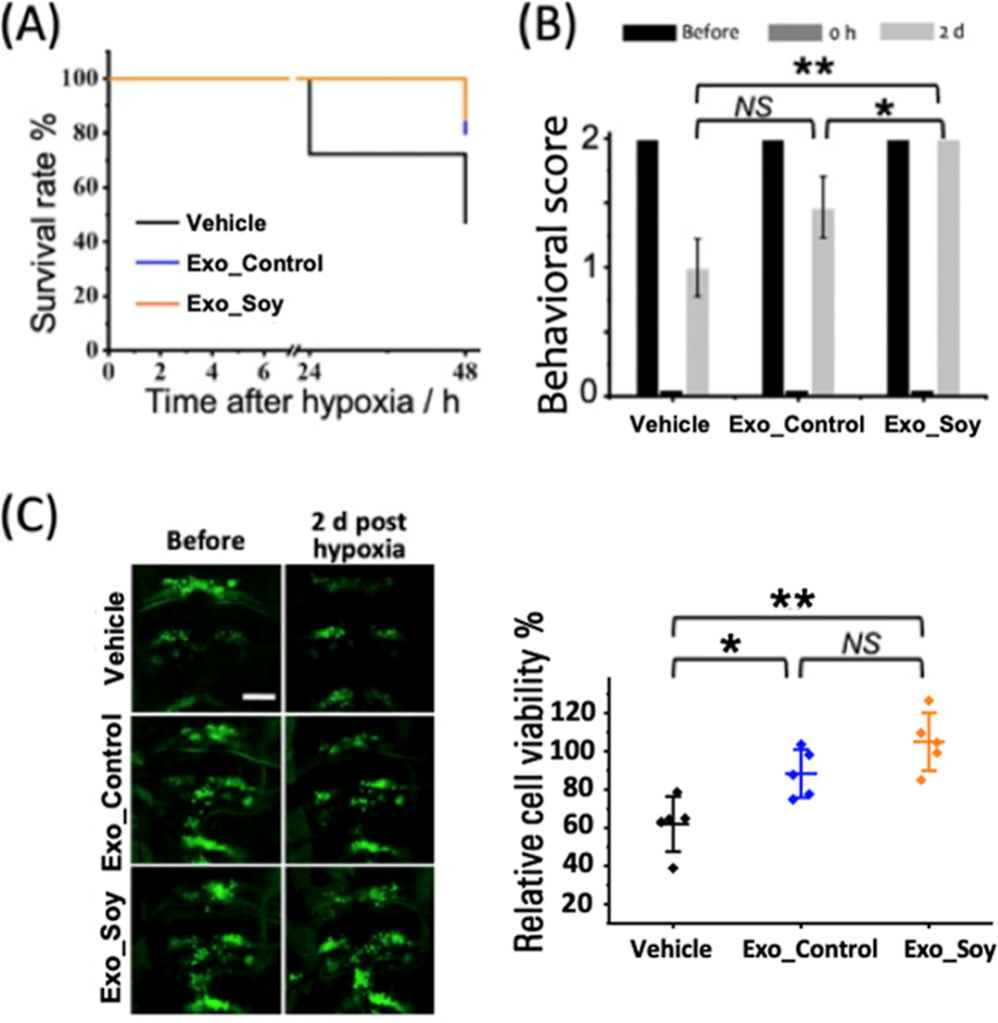
Therapeutic effect of exosomes on larval survival, neurological function, and cerebral cell viability. (A) Survival curve of zebrafish larvae (6 dpf) treated with vehicle, control exosomes (Exo_Control), or soy-derived exosomes (Exo_Soy) posthypoxia. Data represent *n* = 60 larvae per group. (B) Behavioral assessment of neurological function, evaluated immediately after hypoxia (0 h) and 2 days posthypoxia (2 d). Data are presented as mean ± SD from three independent experiments with *n* = 5 larvae per experiment. (C) Left: Representative fluorescence images of cranial motor neurons (green: GFP-labeled) in zebrafish larvae, acquired before and 2 days posthypoxia. Scale bar: 50 μm. Right: Quantification of relative cerebral cell viability at 2 days posthypoxia in the Vehicle, Exo_Control, and Exo_Soy groups. Data are presented as mean ± SD with individual data points (*n* = 5 per group).

To assess the therapeutic efficacy of exosomes in mitigating hypoxia-induced brain injury, we then evaluated the effects of these two exosome treatments on survival, neurological function, and cerebral cell viability in zebrafish larvae following a 15 min hypoxic insult. Figure 4A presents the survival curves of zebrafish larvae (6 dpf) treated with Vehicle, Exo_Control, or Exo_Soy. The Exo_Soy treatment significantly improved survival compared to the Vehicle group, with a slight but noticeable advantage over Exo_Control. At 48 h posthypoxia, survival rates remained above 80% for both Exo_Soy (85 ± 5.8%) and Exo_Control (80 ± 11.5%) groups, whereas the Vehicle group displayed a significantly lower survival rate of 47.2 ± 8.5%.

To further evaluate the therapeutic effects of exosomes, we conducted a behavioral assessment of neurological function. As shown in Figure 4B, Exo_Soy significantly improved neurological function compared with the Vehicle group, with Exo_Control showing intermediate efficacy. Immediately following the hypoxic insult, all larvae exhibited severe neurological deficits (Score = 0). However, by 2 days posthypoxia, larvae treated with Exo_Soy showed substantial recovery in neurological function (Score = 2.0 ± 0), significantly outperforming the Vehicle-treated larvae (Score = 1.0 ± 0.2, *p* < 0.01). Exo_Control-treated larvae demonstrated moderate improvement, with a neurological score of 1.5 ± 0.2, although this difference did not reach statistical significance compared to Vehicle treatment (*p* > 0.05).

To investigate the protective effects of exosomes on cerebral cell viability, we examined GFP-labeled motor neurons in the cranial region. Figure 4C (left panel) shows representative images of neuronal viability 2 days posthypoxia, revealing that Exo_Soy treatment preserved neuronal integrity, as evidenced by stronger fluorescence compared to the Vehicle group. Quantitative analysis in Figure 4C (right panel) indicates that relative cerebral cell viability was significantly higher in the Exo_Soy group (104.9 ± 15.1%) compared to the Vehicle group (62.0 ± 14.4%, *p* < 0.01). Exo_Control treatment also conferred moderate improvement (88.4 ± 15.1%, *p* < 0.05), though no significant difference was observed between the Exo_Control and Exo_Soy groups (*p* > 0.05). These findings suggest that Exo_Soy provides substantial neuroprotection following a 15 min hypoxic insult, as reflected by enhanced survival, improved neurological recovery, and better preservation of cerebral cell viability. While Exo_Control also demonstrated therapeutic benefits, its efficacy was lower compared to Exo_Soy across all measured outcomes.

#### 3.2.2 Exosomes Mitigate Structural and Functional Damage to the NVU Following Hypoxia Insult

**Figure 5:**
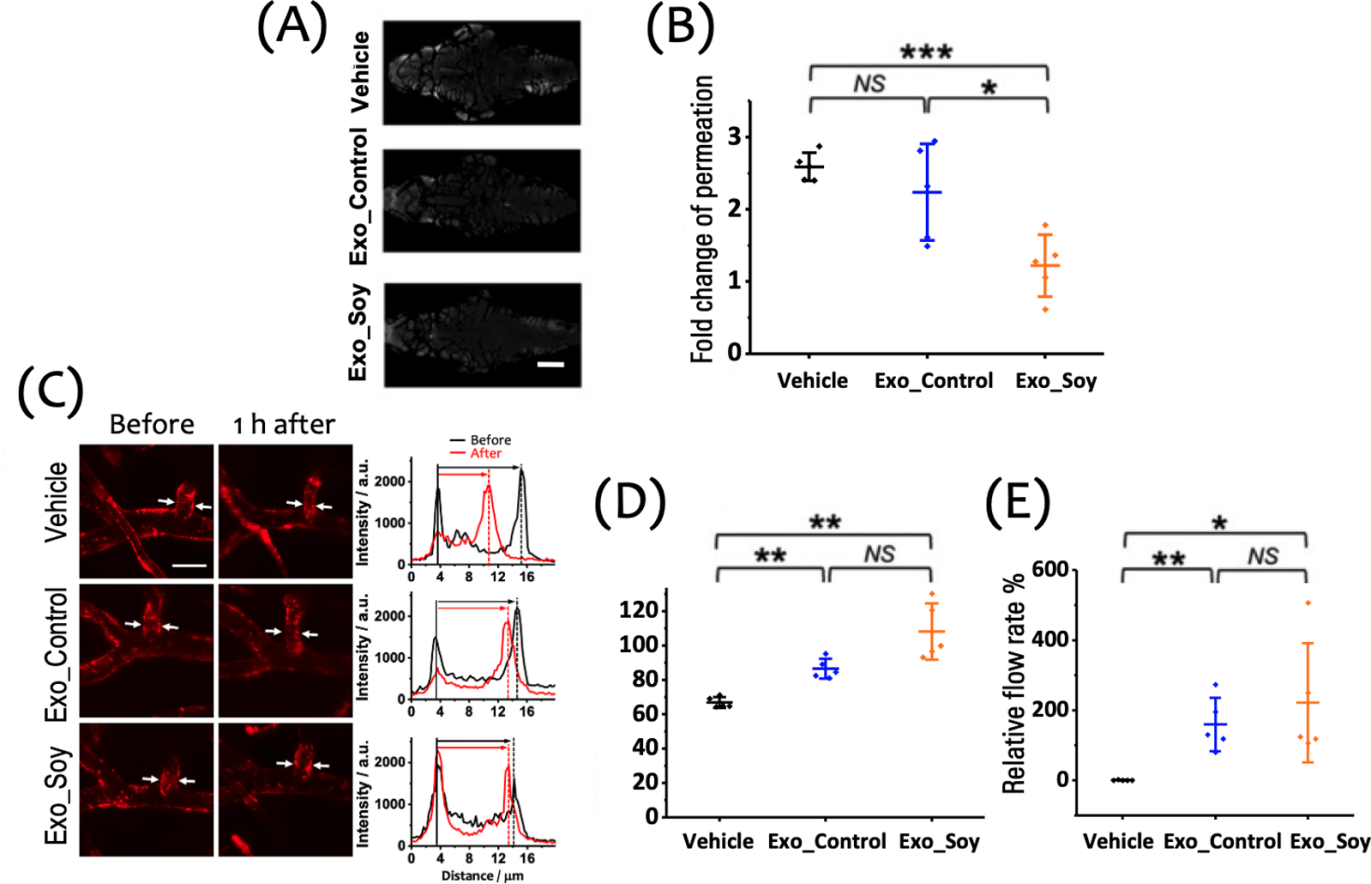
Therapeutic effect of exosomes on the function and integrity of the NVU. (A) Representative images of extravascular fluorescent tracer accumulation in the cranial region of zebrafish larvae 1 h posthypoxia, illustrating the degree of BBB disruption under each treatment condition (gray: RITC-dextran, MW: 10 k). Scale bar: 100 μm. (B) Quantitative analysis of fold change in tracer permeation, indicating BBB integrity across the Vehicle, Exo_Control, and Exo_Soy groups (*n* = 5 per group). (C) Left: Representative images of the cerebral vasculature in zebrafish larvae treated with Vehicle, Exo_Control, or Exo_Soy after a 15 min hypoxic insult (red: mCherry). White arrows point to the regions where vessel width measurements were taken. Right: Cross-sectional fluorescence intensity profiles along the cerebral vessels, illustrating changes before and 1 h after hypoxia in each treatment group. Scale bar: 20 μm. (D) Quantitative analysis of the relative vessel width, showing that Exo_Soy significantly preserves the vessel width compared to the Vehicle group, while Exo_Control displays a similar trend. (E) Quantitative analysis of the relative cerebral blood flow, demonstrating significant improvement in the Exo_Soy group compared to the Vehicle group, with Exo_Control showing comparable efficacy. Data are shown as mean ± SD with individual data points (*n* = 5 larvae per group) in panels (B), (D), and (E).

To assess the therapeutic effects of exosomes on BBB integrity and cerebral vasculature following a 15 min hypoxic insult, we performed imaging and quantitative analysis on zebrafish larvae treated with Vehicle, Exo_Control, or Exo_Soy. We first evaluated BBB integrity using RITC-dextran (MW: 10 k) as a fluorescent tracer. Representative images of tracer accumulation in the cranial region 1 h posthypoxia (Figure 5A) revealed significant extravascular accumulation of the tracer in the Vehicle group, indicating pronounced BBB disruption. In contrast, larvae treated with either Exo_Control or Exo_Soy displayed reduced tracer accumulation, suggesting that exosome treatments helped to maintain BBB integrity under hypoxic conditions. Quantitative analysis (Figure 5B) showed that the Vehicle group had the highest fold change in tracer permeation, reflecting severe BBB damage. Both exosome-treated groups exhibited significantly lower levels of tracer permeation, with Exo_Soy providing the strongest protection. Statistical analysis confirmed that Exo_Soy significantly reduced tracer permeation compared to the Vehicle group (*p* < 0.001) and Exo_Control group (*p* < 0.05), while no significant difference was observed between the Vehicle and Exo_Control groups (*p* > 0.05). These results highlight the superior protective effect of Exo_Soy on BBB integrity.

We next examined the structural changes in cerebral vasculature. Representative images (Figure 5C, left) and cross-sectional fluorescence intensity profiles (Figure 5C, right) demonstrate vessel narrowing following hypoxia in the Vehicle group, indicating vasoconstriction. Both Exo_Control and Exo_Soy treatments mitigated this narrowing effect, helping to preserve the structural integrity of the cerebral vessels. Quantification of the vessel width (Figure 5D) further supports these observations, showing that Exo_Soy treatment significantly preserved the vessel width compared to the Vehicle group (*p* < 0.01), with Exo_Control exhibiting similar effects. No significant difference was observed between Exo_Soy and Exo_Control (*p* > 0.05), suggesting comparable efficacy in preventing vasoconstriction.

Lastly, we assessed the effects of exosome treatments on the cerebral blood flow. As shown in Figure 5E, both Exo_Soy and Exo_Control significantly improved the cerebral blood flow compared to that in the Vehicle group. While Exo_Soy exhibited the highest flow rates, the difference between Exo_Soy and Exo_Control was not statistically significant (*p* > 0.05). Statistical analysis confirmed that the blood flow in the Exo_Soy group was significantly higher than in the Vehicle group (*p* < 0.05), and Exo_Control also showed a significant improvement over the Vehicle group (*p* < 0.01). These findings indicate that both exosome treatments, particularly Exo_Soy, effectively mitigate the structural and functional damage to the NVU caused by hypoxic insult. This is evidenced by enhanced BBB integrity, preservation of the vessel width, and improved cerebral blood flow.

#### 3.2.3 Visualization of Exosome Distribution and Extravasation in a Zebrafish Hypoxic Brain Injury Model

**Figure 6:**
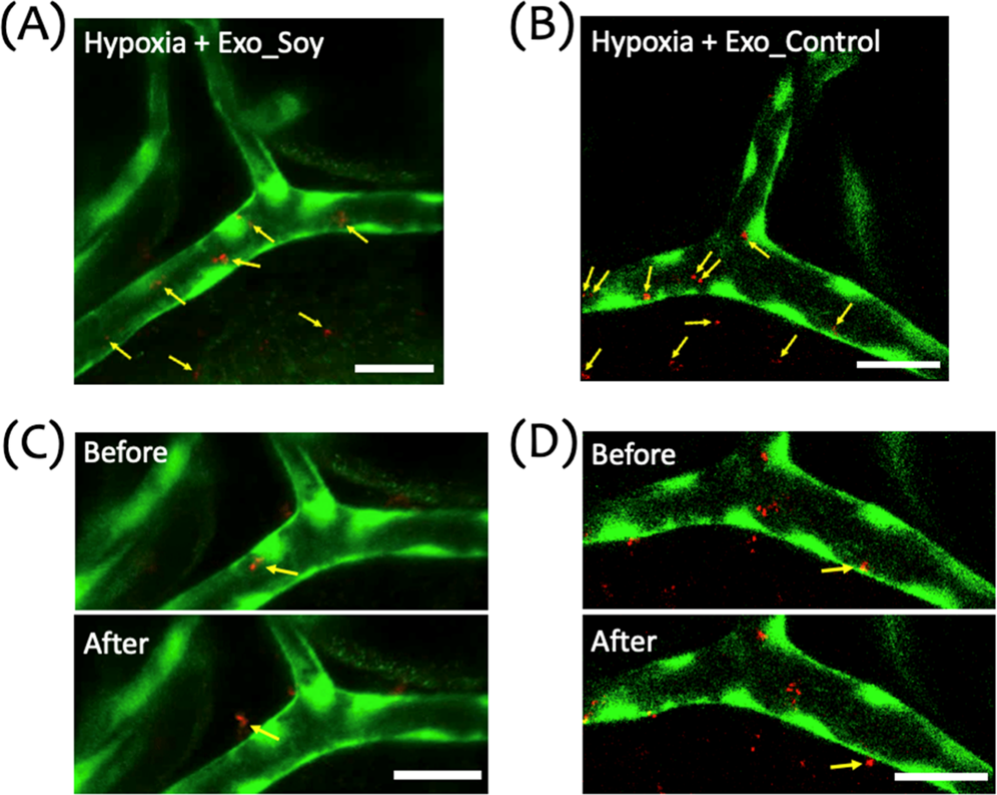
Distribution and extravasation of exosomes in a zebrafish hypoxic brain injury model. (A,B) Representative image of the cranial vasculature in zebrafish larvae subjected to a 15 min hypoxic insult, followed by microinjection of DiI-labeled Exo_Soy (A) or Exo_Control (B) exosomes. The image illustrates the distribution of exosomes within and outside the vasculature. Green represents the vasculature (GFP), and red indicates exosomes (DiI). Yellow arrows point to exosomes located both inside the vessel and those that have extravasated across the vascular wall. (C,D) Sequential images showing exosome migration and extravasation in hypoxia-exposed larvae. Panels illustrate Exo_Soy (C) and Exo_Control (D) before (top) and after (bottom) crossing the vascular wall. Yellow arrows mark exosomes transitioning from intravascular to extravascular regions. Scale bar: 20 μm.

To further explore the behavior of exosomes in the hypoxia-exposed brain, we microinjected DiI-labeled exosomes (Exo_Soy or Exo_Control) into Tg(kdrl) zebrafish larvae (6 dpf) 15 min after the hypoxic insult. This approach allowed for the visualization of exosome distribution and potential extravasation into the brain parenchymal tissue. As shown in Figure 6A,B, both Exo_Soy and Exo_Control exosomes were observed adhering to the vascular wall and extravasating into the surrounding parenchyma (yellow arrows). This suggests that hypoxia promotes conditions that facilitate the adhesion of exosomes to the vascular wall and subsequent extravasation across the compromised BBB. Figure 6C,D presents sequential images illustrating the exosome extravasation process in the hypoxia-exposed group. The top panel captures exosomes localized at the vascular wall prior to extravasation, while the bottom panel shows the migration of exosomes into the parenchymal tissue. This sequence underscores the dynamic nature of exosome extravasation facilitated by BBB impairment due to hypoxia. These findings are consistent with our earlier observations of BBB disruption and increased tracer permeability in hypoxia-exposed larvae. The compromised BBB under hypoxic conditions likely facilitates the extravasation of exosomes from the vasculature into the parenchyma, which may be a critical factor contributing to the therapeutic efficacy of exosomes in mitigating hypoxia-induced brain injury.

#### 3.2.4 Functional Relevance of Exosomal Protein Cargos

**Figure 7:**
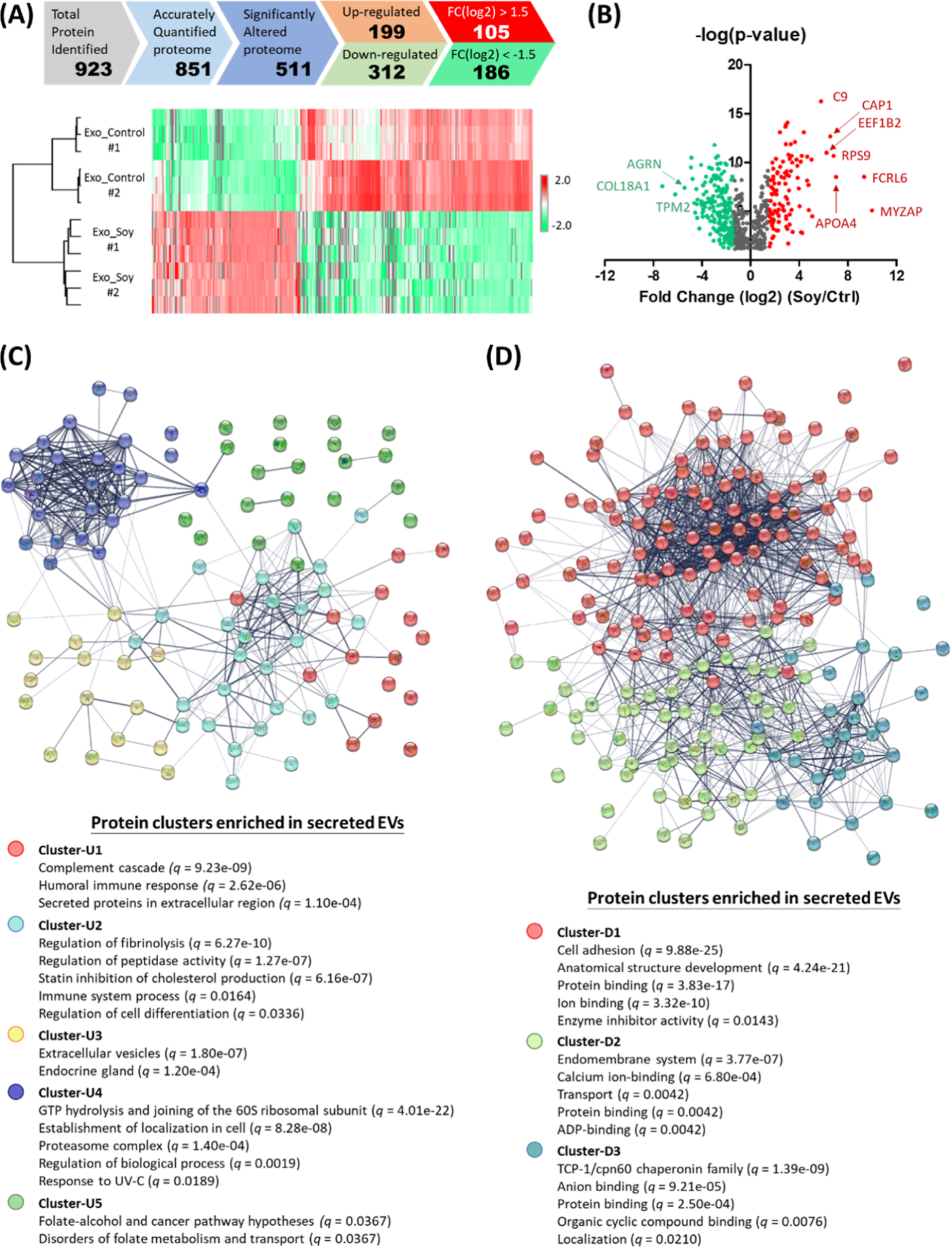
LC–MS/MS proteomic analysis of soy protein-cultured MSC-secreted exosomes. (A) 511 differentially expressed proteins (199 upregulated plus 312 downregulated) were identified by Student’s *t*-test (permutation-based FDR <0.05) (top); reproducibility of the analysis: both control and soy protein-cultured MSC-secreted EV samples were collected within the same batch (*n* = 3) and batches on the different days (#1 and #2) (bottom). (B) Volcano plots of proteins identified in LC–MS/MS. The red and green dots represent the upregulated and downregulated proteins, respectively (*p* < 0.05 and fold change: ±1.5). (C) Upregulated and (D) downregulated proteins were categorized into 5 (up: U1–U5) and 3 (down: D1–D3) functional clusters based on the STRING protein–protein interactome. The top-five key pathways enriched in each cluster with *q*-values less than 0.05 were presented.

To investigate the functional impacts of secreted exosomes, we analyzed the protein composition of their cargos using label-free quantitative proteomic analysis. Two biological replicates of Exo_Soy and Exo_Control were harvested, followed by proteolytic digestion and desalting. Each sample was analyzed with three technical replicates using LC–MS/MS. Among the 923 identified proteins, 851 were quantified at the protein level across all triplicate replicates in at least one biological condition. Statistical analysis (permutation-based FDR <0.05 via student’s *t*-test) identified 511 differentially expressed proteins (199 upregulated and 312 downregulated) (Figure 7A and Table S1). Using log_2_ (fold change) more than 1.5 or less than −1.5 as the cutoffs, we further focused on 105 highly upregulated or 186 downregulated proteins (Figure 7B). The highly upregulated proteins can be categorized into 5 functional clusters (U1 to U5) based on STRING protein–protein interactome (https://string-db.org/) (Figures 7C and S8A). Proteins in clusters U1 and U2 are known secreted proteins involved in immune regulation and cholesterol metabolism, respectively. Several complements (C5, C8b, and C9) and their regulatory proteins were defined in Cluster-U1 (Figure S8B), an equipment that functions as scavengers to fix complements and allows membrane attack complex (MAC) formation on the EV surfaces, thus subsequently protecting the cells or tissues from complement attack and alleviating inflammation responses.^34,35^ Several apolipoproteins (APOs), including APOA4, APOC3, APOE, APOB, and APOH, were found to form the core network of Cluster-U2 that participates in chylomicron remodeling and VLDL clearance, leading to a reduction of cholesterol levels in the local microenvironment (Figure S8C). In addition, those APOs, in collaboration with several peptidases, can regulate coagulation signaling and fibrinolysis, thus controlling blood clotting. With the connection with the complement cascades in Cluster-U1, some proteins in Cluster-U2 also play roles in regulating immune responses. Cluster-U3 contains proteins routinely detected in secreted exosomes from endocrine glands (Figure S8D). Notably, several ribosomal proteins (RPs) and RNA-binding proteins (RBPs) in Cluster-U4 form the interactome with proteins involved in the regulation of biological processes, including response to UV–C exposure, intracompartmental localization, and protein turnover in the proteasome complex (Figure S8E). The presence of RPs and RBPs has been recently suggested as the key mechanism to package RNA into exosomes known as exosomal-shuttle RNA (esRNA) for cell–cell communication and genetic exchange between cells, sometimes can even change the phenotypes of recipient cells.^36−38^ Proteins in Cluster-U5 are the ones with an unidentified functional network. Some may play roles in folate–alcohol metabolism and transport (Figure S8F).

On the other hand, the highly downregulated proteins can be categorized into 3 clusters (D1 to D3) (Figures 7D and S9A). Cluster-D1 is the major group containing 102 proteins involved in ECM-mediated cell adhesion and tissue development, suggesting a microenvironment to maintain cells at a stem-cell-like stage (Figure S9B). Proteins in Cluster-D2 are related to endomembrane and intracellular vesicle transportation systems (Figure S9C). Although the detailed mechanisms remain unclear, several lines of evidence suggest a crosstalk between exosome biogenesis and autophagy, which can influence cell death in recipient cells by disrupting the balance of key components required for autophagosome formation.^39,40^ In Cluster-D2, CLTC, a critical scaffolding protein in the fusion of the autophagosome with other vacuoles, was significantly downregulated. Additionally, several proteins associated with ER stress-induced cell death, including PDIA3, PDIA6, CALR, HYOU1, CANX, and DNAJB11, were also downregulated (Figure S10). Furthermore, key components involved in melanosome formation, which are believed to function as specialized autolysosomes that accumulate undegraded proteins and lipids in the aging human brain, particularly in patients with Parkinson’s disease,^41,42^ were downregulated in Cluster-D2. These findings related to proteins in Cluster-D2 suggest a reduction in oxidative stress and neuroinflammation in the microenvironment, which may promote cell survival under stresses.

In Cluster-D3, a TCP-1/cpn60 chaperonin complex was found in the core of the interactome, which is responsible for protein folding, DNA/RNA binding, and assembly of telomerase and ribosome complexes, thus determining protein translation and metabolism (Figure S9D). Increased levels of components in the TCP-1/cpn60 chaperonin complex in exosomes derived from tumor cells, such as CCT3, CCT6A, and TCP1, have been linked with cancer aggressiveness.^43,44^ Interestingly, a previous study indicated the ability of the TCP-1/cpn60 chaperonin complex to act as an intercellular signal that stimulates immune cells to produce proinflammatory cytokines.^45^ Reduced levels of this protein network in the secreted exosomes may imply the benefits of reduced immune responses in the local microenvironment.

## 4 Discussion

Therapeutic options for hypoxia-induced brain injury remain limited, highlighting the need for novel therapeutic developments. Using mammals for drug screening can be costly and raises ethical concerns, particularly regarding animal rights. To address this, we employed a zebrafish hypoxia-induced brain injury model, which exhibits phenotypes similar to those observed in mammal models. This model is suitable for a phenotype screen and image-based quantitative assessment, making it a valuable tool for identifying novel therapeutic strategies. Previous studies have shown that hypoxia can damage vascular endothelial cells, leading to a loss of BBB integrity in humans and experimental animal models.^2,7^ Survival from hypoxia-induced brain injury also revealed severe neurological impairment.^17^ Similarly, our zebrafish model demonstrated cerebral cell death and impaired neural function (Figure 2), along with decreased cerebral blood flow and a clear sign of BBB damage (Figure 3), following hypoxic insult.

In this study, we further show that exosome therapy holds therapeutic potential in ameliorating hypoxia-induced brain injury, underscoring the utility of our zebrafish model for a phenotype-based screen of treatments targeting such injuries. Exosomes secreted from WJ-MSCs cultured on soy protein-coated surfaces (Exo_Soy) showed superior therapeutic efficacy in most evaluated outcomes compared to the control group (Exo_Control). Notably, Exo_Soy treatment led to significant improvements in maintaining neurological function (Figure 4B) and preserving BBB integrity (Figure 5A,B). Moreover, Exo_Soy decreased cerebral cell death (Figure 4C) and mitigated cerebral vasoconstriction, resulting in enhanced cerebral blood flow being restored (Figure 5C,E). To confirm that the effect was not simply owing to the released soy proteins, the soy protein-coated plate was also incubated with the culture medium without WJ-MSCs (i.e., Medium (Soy)) and then treated in the hypoxia-induced injury zebrafish model. As shown in Figure S11, an evaluation of the survival rate and neurological function of the larvae revealed that Medium (Soy) did not demonstrate any discernible advantages. Using real-time confocal imaging to track exosomes in living zebrafish, we observed that these exosomes were able to extravasate across the cerebral vessel wall (Figure 6), strongly indicating that the compromised BBB under hypoxic conditions likely facilitated their permeation into the brain parenchyma, contributing to their therapeutic efficacy in mitigating hypoxia-induced brain injury.

Based on the protein–protein interactome study on differentially expressed protein cargos in the Exo_Soy, several lines of evidence suggest the prospects of those exosomes as potential therapeutics to target diseases associated with NVU.^34^ First, they protect cells/tissues from the attacks of complements (humoral immunity; Cluster-U1) and immune cells (cell-mediated immunity; Cluster-D1 and D3). With the reduction of ER-mediated stress and autophagosome activity (Cluster-D2), the local inflammation and oxidative stresses can be further suppressed, leading to better survival advantages for the recipient cells. Those findings support previous studies that MSC-derived exosomes can significantly reduce the levels of C3 in the brain or blood of treated mice.^46^ Reduction of MAC formation and pro-inflammatory cytokines after exosome treatments can suppress the activation of B cells^47^ and neutrophils,^48^ as well as the phenotype transition into an inflammatory state in microglia^47^ or a neurotoxic state in astrocytes.^49^ On the other hand, the findings of increased RPs and RBPs in the exosomes (Cluster-U4) indicate the potential benefits of esRNAs in protecting against vascular and neurological diseases. Finally, the upregulation of certain APOs (Cluster-U2) provides several benefits in VLDL/LDL clearance and vascular health by suppressing blood clotting. To our knowledge, this is the first report to indicate increased levels of APOs in MSC-derived exosomes enriched by our unique soy protein culture. Current results raise again a continuously discussed question about the classification of proteins such as APOB, ALB, or complement proteins as active and functional integral components of the protein corona that surrounds exosomes or the contaminants that should be removed.^50,51^ Our obtained Exo_Soy was of a larger diameter compared to that of Exo_Control (Figure 3C). The diameter changes potentially indicated the protein corona differed.^52^ Despite most research conducted on protein corona in exosomes derived from the blood plasma or serum, it is evident that a corona can also form on the exosome surface in a cell culture, given the presence of diverse proteins in the media that can bind through electrostatic or affinity interactions. Besides, the presence of different nutrients may also trigger different cell responses and the resulting secreted EVs. Consequently, it is reasonable to expect that cell culture with the soy protein will also exhibit a distinctive profile of exosomal proteins. While detailed mechanisms required further validation, our results undoubtedly demonstrated that the potential of exosomes derived from the soy protein culture could effectively suppress hypoxia-induced brain damage, possibly by ameliorating neurovascular abnormality.

The zebrafish model provides significant advantages for studying therapeutic interventions for hypoxia-induced brain injury including high fecundity, optical transparency, and cost-effectiveness. It recapitulates key phenotypes observed in mammals, such as BBB disruption, reduced cerebral blood flow, and neural dysfunction, making it valuable for phenotype-based therapeutic screening. However, zebrafish lack a fully developed immune system and have a simpler vasculature, which may influence exosome interactions with the BBB and limit direct translation to mammalian systems. Differences in metabolic rates and molecular pathways further necessitate caution in generalizing the findings. Nevertheless, zebrafish offer a complementary, high-throughput platform for preclinical studies with future validation in mammalian models needed to ensure translational relevance.

## 5 Conclusions

Our work significantly advances the use of the zebrafish model for phenotype-based assessment of novel therapies, especially when targeting the NVU. The model closely replicates key features of its mammalian counterparts, including vasoconstriction, BBB disruption, impaired cerebral perfusion, cerebral cell death, and neurological deficit. Furthermore, we demonstrated that the therapeutic potency of WJ-MSC-secreted exosomes could be enhanced by simply culturing cells on the soy protein-coated surface. This finding shows that simple nutrient modifications, such as introducing soy proteins, can significantly alter exosomal cargos, providing a new perspective on exosome research and therapeutic applications. Recent studies implied that nanotopography mattered in regulating EVs.^23^ Although in the current study, we coated soy proteins directly on the culture plate, it will also be interesting to fabricate soy protein films to fine-tune their roughness and stiffness or to graft additional molecules, altering the hydrophobicity to explore the resulting effects on MSC-secreted exosomes as the physicochemical properties of the extracellular matrix potentially affect the cargo sorting of various biomolecules into EVs, regulating their delivery.^53^ Besides, future studies should focus on systematically optimizing exosome doses (in a preliminary tube formation assay, we have observed a dose-dependent effect of these exosomes (Figure S12)). Further validating the identified interactomes and pinpointing the specific molecules responsible for facilitating exosome permeation into the brain parenchyma will be also essential. Since the protein corona surrounding exosomes can influence their physicochemical properties, cellular uptake, and in vivo distribution,^51,54^ a deeper understanding of the dynamic interactions between these proteins and the microenvironment will enable better manipulation of the exosomal protein corona for more efficient targeting and improved therapeutic outcome.^55,56^

## Supporting Information Available

Additional experimental details about zebrafish model setup, in-solution protein digestion and LC–MS/MS measurement and toxicity assessment of WJ-MSC-secreted exosomes, STRING protein–protein interaction analysis, and control experiments (PDF)
A list of differentially expressed MS-identified proteins (XLSX)
