# Supplementary material for: Soy Protein-Cultured Mesenchymal Stem Cell-Secreted Extracellular Vesicles Target the Neurovascular Unit: Insights from a Zebrafish Brain Injury Model
Source: ACS Biomater Sci Eng. 2025 Feb 25;11(3):1432–44. doi: 10.1021/acsbiomaterials.4c02304 (PMC11897944; doi:10.1021/acsbiomaterials.4c02304)
Supplement: Supplementary file 1 — ab4c02304_si_001.pdf [file ab4c02304_si_001.pdf]

Supporting Information

Soy protein-cultured mesenchymal stem cell-secreted extracellular vesicles target the neurovascular unit: Insights from a zebrafish brain injury model

Tai-I Lin[#,1], Pei-Ying Hsieh[#,1], Hui-Jen Lin[1], Cheng-Kang Chiang[2], Jim Jinn-Chyuan Sheu[3], Wei-Tien Chang[4], Ian Liau[1,5,*] and Hsin-Yun Hsu[1,5,*]

[1]Department of Applied Chemistry and Institute of Molecular Science, National Yang-Ming Chiao-Tung University, Hsinchu 300093, Taiwan.
[2]Department of Chemistry, National Dong Hwa University, Hualien 974301, Taiwan.
[3]Institute of Biomedical Sciences, National Sun Yat-Sen University, Kaohsiung 804201, Taiwan.
[4]National Taiwan University Hospital/National Taiwan University, Taipei 100233, Taiwan.
[5]Center for Emergent Functional Matter Science, National Yang-Ming Chiao-Tung University, Hsinchu 300093, Taiwan.

[#] T.-I. Lin and P.-Y. Hsieh contributed equally to this work.

*Corresponding authors:
hyhsu99@nycu.edu.tw (H.-Y. Hsu) and ianliau@nycu.edu.tw (I. Liau),
Department of Applied Chemistry and Institute of Molecular Science, National Yang-Ming Chiao-Tung University, No.1001 Ta-Hsueh Road, Hsinchu 300093, Taiwan.
Center for Emergent Functional Matter Science, National Yang-Ming Chiao-Tung University, No.1001 Ta-Hsueh Road, Hsinchu 300093, Taiwan.

1. Supplementary materials and methods

Chemicals. 1-Phenyl 2-thiourea (PTU), tricaine, sodium chloride (NaCl), potassium chloride (KCl), calcium chloride ($CaCl_2$), magnesium sulfate ($MgSO_4$), methylene blue, bovine serum albumin (BSA), urea, sodium deoxycholate, sodium lauroyl sarcosinate, ammonium bicarbonate, dithiothreitol, iodoacetamide and glycerol were obtained from Sigma-Aldrich. Isoflurane was sourced from Alfa Aesar, while Rhodamine B isothiocyanate–Dextran (10 kDa) was purchased from Merck. The lipophilic tracer DiI and DiD was supplied by Invitrogen/Thermo Fisher. Minimum Essential Medium (MEM), fetal bovine serum (FBS), non-essential amino acids (NEAA) 100× solution, and sodium pyruvate 100× solution, and antibiotic-Antimycotic solution were procured from Gibco™-Thermo Fisher Scientific Inc. Soy protein isolate (SPI) was provided by Archer Daniels Midland, USA. Protease inhibitors and phosphatase inhibitors (EDTA-free) were purchased from Roche.

Animal handling. Animal handling procedures were approved by the Animal Investigation Committee of National Yang-Ming Chiao-Tung University (Approval number: 1101252). Four transgenic zebrafish lines, AB, Tg(CM-isl1:GFP) [1], Tg(kdrl:mCherry) [2], Tg(kdrl:EGFP) [3], and Tg(gata1:DsRed) strains [4], were obtained from the Taiwan Zebrafish Core Facility for use in this study. The Tg (CM-isl1:GFP) strain expresses GFP in cranial motor neurons, Tg (kdrl:mCherry) strain expresses mCherry in the vascular endothelium, Tg (kdrl:EGFP) strain expresses EGFP in the

---

[1] S.-i. Higashijima, Y. Hotta and H. Okamoto. Visualization of cranial motor neurons in live transgenic zebrafish expressing green fluorescent protein under the control of the Islet-1 promoter/enhancer. *The Journal of Neuroscience* **20**, 206 (2000).

[2] E. Y. N. Lam, C. J. Hall, P. S. Crosier, K. E. Crosier and M. V. Flores. Live imaging of Runx1 expression in the dorsal aorta tracks the emergence of blood progenitors from endothelial cells. *Blood* **116**, 909 (2010).

[3] S.-W. Jin, D. Beis, T. Mitchell, J.-N. Chen and D. Y. R. Stainier. Cellular and molecular analyses of vascular tube and lumen formation in zebrafish. *Development* **132**, 5199 (2005).

[4] D. Traver, B. H. Paw, K. D. Poss, W. T. Penberthy, S. Lin and L. I. Zon. Transplantation and in vivo imaging of multilineage engraftment in zebrafish bloodless mutants. *Nature Immunology* **4**, 1238 (2003).

vascular endothelium, and Tg (gata:DsRed) strain expresses DsRed in red blood cells (RBCs), respectively. Adult and larval zebrafish were maintained according to standard protocols. Larvae at six days post-fertilization (dpf) were used for all experiments in this study.[5]

Hypoxia induction and resuscitation. To model asphyxia and resuscitation in larval zebrafish, we developed a system comprising two chambers: a hypoxia chamber and a normoxia chamber. This setup allowed for precise and consistent exposure to hypoxic conditions for controlled durations, followed by rapid reoxygenation in a normoxic environment.

The hypoxia chamber was prepared by infusing pure nitrogen gas into the water at a flow rate of 6 L/min for 30 minutes to displace dissolved oxygen. After this initial period, the flow rate was reduced to 2 L/min to maintain hypoxic conditions. Throughout the experiments, the dissolved oxygen (DO) concentration was sustained below 0.2 mg/L, while the temperature was maintained at approximately 28.5 °C. According to the guidelines set by the Commission on the Environment and Natural Resources, DO levels below 2 mg/L are classified as hypoxic. The normoxia chamber was filled with aerated system water, ensuring a DO concentration of approximately 7.5 mg/L at 28.5 °C, which represents standard oxygen levels for zebrafish larvae.

To assess hypoxia-induced injury, zebrafish larvae were randomly assigned to one of three groups: Hypoxia, Sham, and Control. For the Hypoxia group, larvae were placed in the hypoxia chamber for the designated hypoxic duration and then immediately transferred to the normoxia chamber to simulate resuscitation. The Sham group larvae were placed in the normoxia chamber for an equivalent duration and

[5] K.-Y. Lin, W.-T. Chang, Y.-C. Lai and I. Liau. Toward functional screening of cardioactive and cardiotoxic drugs with zebrafish in vivo using pseudodynamic three-dimensional imaging. *Analytical Chemistry* **86**, 2020 (2014).

subjected to the same handling procedures as the Hypoxia group but without exposure to hypoxia, thereby controlling for any stress related to handling and chamber transfers. The Control group remained undisturbed in the normoxia chamber throughout the entire experiment.

Determination of survival curve. To assess larval survival rates, we inspected the zebrafish larvae using a stereomicroscope (SMZ 645, Nikon) at designated time points post-hypoxia (Figure S1). Larvae were considered dead if they exhibited cardiac arrest or a loss of body pigmentation (albino appearance). The survival rate at each time point was calculated using the following formula:

$$Survival\ rate\ at\ a\ designated\ time\ point = (B/A) \times 100\%$$

where A represents the initial number of larvae in the group, and B denotes the number of surviving larvae at the designated time point.

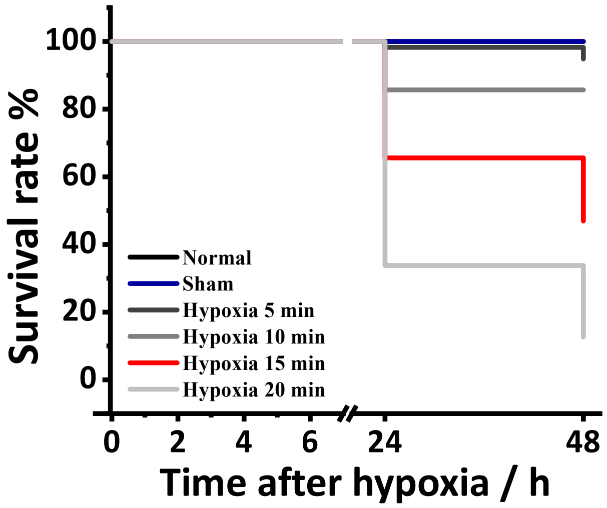

Figure S1 Survival rates of zebrafish larvae (6 dpf) following hypoxic exposure for 5, 10, 15, and 20 minutes. The survival curves show a gradual decline in survival with longer hypoxic durations. The 15-minute hypoxia group, which resulted in an end-point survival rate of 47% (red line), was selected for further experiments as it represents a balance between injury severity and survivability, providing an appropriate model for studying hypoxic-ischemic brain injury and testing therapeutic interventions.

Evaluation of neurological deficit. To assess neurological deficits in larval zebrafish, we developed a behavioral scoring system consisting of two assessments: balance maintenance and response to tactile stimuli.

First, we evaluated each larva's ability to maintain balance over a 30-second period. Larvae that failed to maintain balance were assigned a score of 0, while those that successfully maintained balance received a score of 1. Next, we assessed the larva's response to a stimulus. For each test, a single larva was placed into a plate. The plate was then positioned inside a sound-proof, light-shielded box for at least five minutes to minimize environmental interference and allow the larva to acclimate. After this period, the corner of the plate was gently tapped with a plastic rod, and the larva's behavior was recorded using a digital microscope. Larvae that remained motionless with no response to the stimulus were given a score of 0, while those that exhibited any movement in response were assigned a score of 1.

Determination of cerebral cell viability. To assess the viability of cerebral cells in larval zebrafish, we utilized the transgenic zebrafish line Tg(CM-isl1), which specifically expresses green fluorescent protein (GFP) in motor neurons. Live cells expressing GFP exhibit strong green fluorescence, which diminishes upon apoptotic or necrotic cell death. This allowed for the visualization and quantification of viable cerebral cells based on GFP fluorescence intensity, providing a reliable marker for cell viability.

Imaging was performed using a laser scanning confocal microscope (TCS SP5 II, Leica Microsystems). Prior to imaging, zebrafish larvae were anesthetized with 100 ppm tricaine and immobilized on the microscope stage. For each larva, we acquired 40 to 60 z-stack images at 2 μm intervals with an excitation wavelength of 488 nm and an emission detection range of 495–570 nm. A 20× objective lens (HC PL Apo CS 20×/0.7, Leica) was used to focus on the cranial region, enabling detailed visualization

of cerebral cells.

The protocol for image analysis is outlined in Figure S2. First, a maximum-intensity projection (MIP) image was generated from the z-stack of 2D images using ImageJ software (National Institutes of Health). To isolate regions of interest representing areas of fluorescence, we applied a designated threshold value to the MIP image to create a binarized image. This binarized image was used to determine the area of fluorescence by highlighting pixels above the threshold. Subsequently, a masked image was produced by multiplying the binarized image with the original MIP image. This process allowed us to focus on the fluorescence intensity within the specified areas, effectively isolating the signal from background noise. Cerebral cell viability was quantified by calculating the area-weighted GFP intensity (I), defined by the equation:

$$I = I_{masked} \, / \, I_{binary}$$

In this equation, I_{masked} represents the total fluorescence intensity within the masked image, and I_{binary} denotes the total area (in pixels) of the binarized image. By dividing I_{masked} by I_{binary} we obtained the area-weighted GFP intensity, providing a quantitative measure of cerebral cell viability.

To evaluate cerebral cell damage, we analyzed the larvae both before and 48 hours after exposure to hypoxia. The relative cell viability for each larva was determined by comparing the area-weighted GFP intensity measured at these two time points. The relative cell viability was calculated using the following formula:

$$Relative\ cell\ viability\% = \frac{I_{after}}{I_{before}} \times 100\%$$

where I_{before} and I_{after} represent the area-weighted GFP intensity before and 48 hours after the hypoxic insult, respectively.

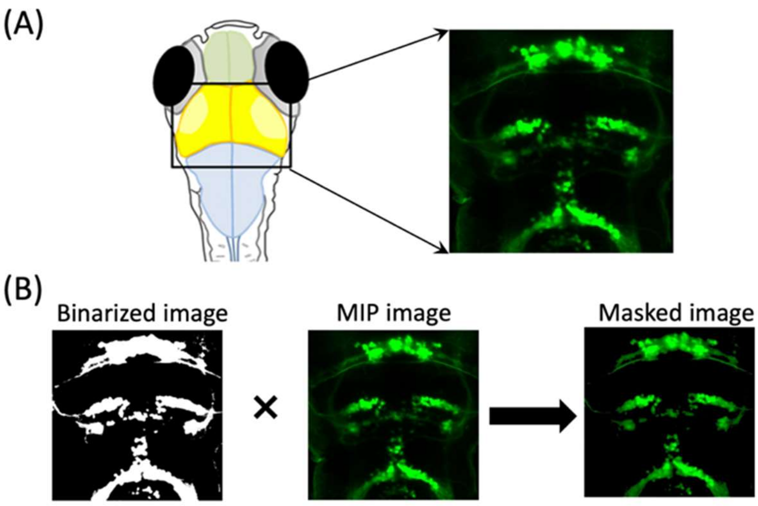

Figure S2 Illustration of imaging-based analysis of cerebral cell death. (A) Schematic representation of the top view of the cranial region of a larval zebrafish, with a rectangle indicating the specific area selected for imaging. (B) Workflow for image analysis: A maximum-intensity projection (MIP) image (middle) was generated from the z-stack of confocal images. A binarized image (left) was created by applying a designated threshold to the MIP image to isolate regions of interest. The binarized image was then multiplied by the MIP image to produce a masked image (right), highlighting the areas used for quantifying cerebral cell viability based on GFP fluorescence intensity.

Determination of the severity of blood-brain barrier (BBB) impairment. The extravascular leakage of fluorescent tracers serves as an indicator of trans-endothelial permeation, providing a quantitative measure of BBB damage. To specifically visualize extravascular regions, we utilized the transgenic zebrafish line Tg(kdrl), which expresses enhanced green fluorescent protein (EGFP) in vascular endothelial cells, thereby delineating the cerebral vasculature.

Prior to imaging, a fluorescent tracer solution consisting of rhodamine isothiocyanate (RITC)-dextran (molecular weight 10,000; 500 μM in 0.5 μL) was microinjected into the cardinal vein (CV) of each larva using a microinjector (PCO-1500, ZGB Pico Liter Microinjector, Genes). One hour post-injection, we acquired 80 to 100 z-stack images of the cranial region using a laser scanning confocal microscope. Imaging parameters were set to capture both the fluorescent tracer and the cerebral vasculature: the tracer was excited at 561 nm with emission collected from 580 to

700 nm, and the EGFP-labeled vasculature was excited at 488 nm with emission collected from 495 to 570 nm. A 10× objective lens (HCX PL APO 10×, N.A. 0.4; Leica) was used for image acquisition.

Figure S3 illustrates the protocol for determining the severity of BBB impairment. First, an MIP image of the fluorescent tracer was generated from the z-stack using ImageJ software. Next, a binarized image of the cerebral vasculature was created by applying a threshold to the EGFP channel. To highlight the extravascular regions, we inverted the binarized vasculature image to produce an extravascular mask. The masked image was then obtained by multiplying the extravascular mask with the MIP image of the fluorescent tracer. This process isolated the fluorescent signal in the extravascular space, allowing for quantification of tracer leakage.

The area-weighted extravascular leakage (I) was calculated using the equation:

$$I = I_{masked} \, / \, I_{binary}.$$

where I_{masked} represents the total fluorescence intensity of the tracer in the extravascular region, and I_{binary} denotes the total area (in pixels) of the extravascular region.

The severity of BBB damage in each larva was quantified by calculating the fold change in permeation relative to the control group, using the formula:

$$Fold\ change\ in\ permeation = I_{exp} \, / \, I_{nl(mean)}$$

where I_{exp} is area-weighted extravascular leakage in the experimental group, and $I_{nl(mean)}$ is the mean area-weighted extravascular leakage in the normal (control) group.

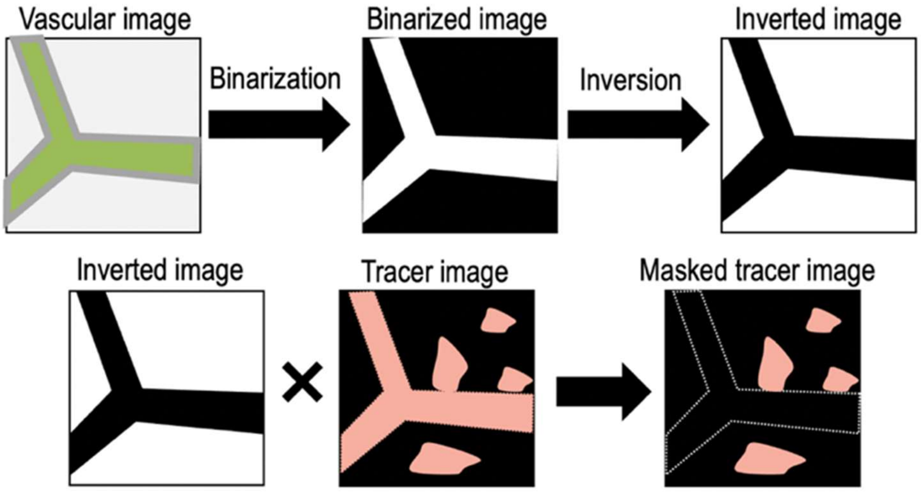

Figure S3 Illustration of imaging-based analysis of BBB impairment. The process begins with the cerebral vasculature image acquired from the EGFP channel, which is binarized to create a black-and-white representation of the vasculature. This binarized image is then inverted to highlight the extravascular space, producing the extravascular mask. The extravascular mask is multiplied by the MIP image of the fluorescent tracer to generate the masked tracer image. This masked tracer image isolates areas of tracer extravasation, indicating BBB impairment.

Determination of changes in cerebral vessel width. To assess changes in cerebral vessel width, we utilized the transgenic zebrafish line, Tg (kdrl:mCherry), which expresses the fluorescent protein mCherry in vascular endothelial cells. This method was adapted from previous studies [6].

First, we identified the first branch of the downstream central artery (CtA1st) using the method described earlier. We then acquired 25 to 30 z-stack images of the CtA1st region using a laser scanning confocal microscope. Imaging parameters were set with an excitation wavelength of 561 nm and an emission detection range of 580–700 nm. Images were captured at 2 μm intervals using a 40× objective lens (HC PL APO 40×, N.A. 0.85; Leica).

[6] P.-T. Kao, I.-J. Lee, I. Liau and C.-S. Yeh. Controllable NO release from Cu1.6S nanoparticle decomposition of S-nitrosoglutathiones following photothermal disintegration of polymersomes to elicit cerebral vasodilatory activity. *Chemical Science* **8**, 291 (2017).

An MIP image was generated from the z-stack. A cross-sectional line was then drawn across the CtA1st near the branch stem, as illustrated in Figure S4A. The resulting cross-sectional fluorescence intensity profile typically displayed two peaks corresponding to the inner walls of the vessel. The vessel width was determined by measuring the distance between these two peaks (Figure S4B).

Vessel width measurements were taken before and one hour after hypoxia exposure for each larva. The relative vessel width was calculated using the following formula:

$$Relative\ vessel\ width\% = W_{after} \,/\, W_{before} \times 100\%$$

where W_{before} and W_{after} represent the vessel width measured before and after hypoxia, respectively.

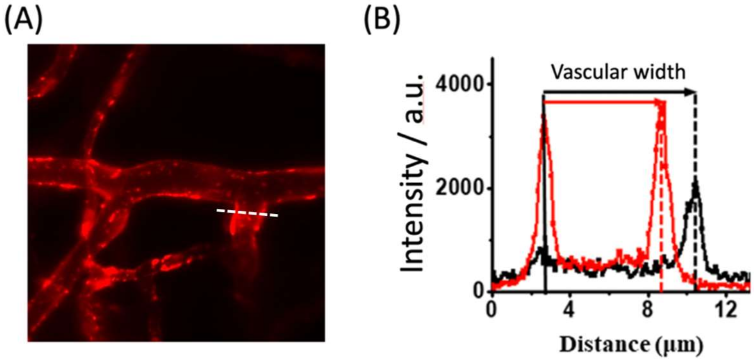

Figure S4 Illustration of imaging-based analysis of cerebral vessel width. (A) A representative MIP image of the region of interest (ROI), highlighting the basilar artery (BA) and the CtA1st (red fluorescence from mCherry expression). To determine the vascular width of CtA1st, a dashed line is drawn across a selected region of the vessel near the branch stem. This line is used to generate a cross-sectional fluorescence intensity profile. (B) A representative cross-sectional fluorescence intensity profile corresponding to the dashed line in (A). The distance between the two intensity peaks represents the inner walls of the vessel. The measured distance between these peaks corresponds to the vessel's width.

Determination of changes in cerebral vessel blood flow rate. To assess changes in cerebral blood flow rate, we measured the number of red blood cells (RBCs) passing through a specific cerebral vessel within one minute. To enable simultaneous fluorescence imaging of both the vasculature and RBCs, we generated a transgenic

zebrafish line expressing EGFP in vascular endothelial cells and DsRed in RBCs by crossing Tg(kdrl) and Tg(gata1) lines.

First, we identified the CtA1st using a three-dimensional (3D) confocal image of the cerebral vasculature as illustrated in Figure S5A. Imaging was performed using a laser scanning confocal microscope with the following settings: Channel 1 for EGFP (λ_{ex}= 488 nm; λ_{em}= 495 ~ 550 nm) and Channel 2 for DsRed (λ_{ex}= 561 nm; λ_{em}= 580 ~ 700 nm). A 40× objective lens (HC PL APO 40×, N.A. 0.85, dry; Leica) was used for imaging.

Next, we conducted line scanning (x–t imaging) perpendicular to the CtA1st for one minute at a scanning rate of 1,000 Hz. This high-speed scanning allowed us to capture the movement of RBCs through the vessel over time. Paired x–t images were generated for both the EGFP fluorescence from the endothelium and the DsRed fluorescence from the RBCs (Figure S5B, left panels). The overlaid x–t images displayed red streaks corresponding to RBCs moving through the vessel.

Individual RBCs were manually counted by identifying the red fluorescent streaks in the overlaid x–t images (Figure S5B, right panel). The number of RBCs counted over the one-minute period represented the blood flow rate for that vessel.

The relative blood flow rate was calculated using the following equation

$$Relative\ RBCs\ flow\ rate\% \ = R_{after}\ /\ R_{before}\ \times\ 100\%$$

where R_{before} and R_{after} represent the number of RBCs passing through the CtA1st before and after hypoxia exposure, respectively.

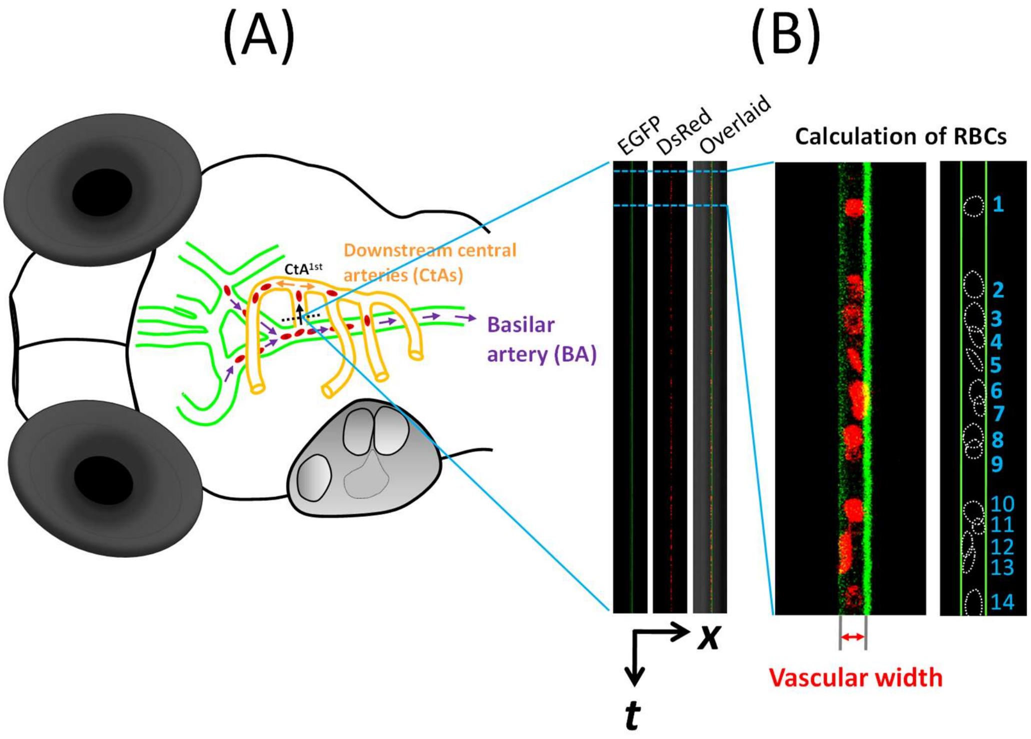

Figure S5 Illustration of imaging-based analysis of cerebral blood flow rate. (A) A schematic representation of the cerebral vasculature, highlighting the downstream CtA1st, which was selected for measuring cerebral blood flow rate. (B) A series of x–t images displaying the vessel wall (green: EGFP) and individual RBCs (red: DsRed). The left panels show the EGFP channel, the DsRed channel, and the overlaid image, respectively. Enlarged views are provided to highlight details. RBCs in the overlaid x–t images were manually identified and counted to determine the blood flow rate, defined as the number of RBCs passing through the vessel in one minute.

In-solution protein digestion. For protein digestion, 100 µg of exosome pellets (Exo_Soy and Exo_Control (n=2 each)) were first resuspended in lysis buffer comprised of 8 M urea, 12 mM sodium deoxycholate, 12 mM sodium lauroyl sarcosinate, and 50 mM ammonium bicarbonate (ABC, pH 8.2), along with EDTA-free protease inhibitors and phosphatase inhibitors. After each sample was reduced and alkylated with 5 mM dithiothreitol (37°C for 30 min) and 10 mM iodoacetamide (room temperature for 30 min in the dark), the samples were diluted with one volume of ABC buffer prior to adding Lys-C at a 1:100 (w/w) ratio and incubated at 37 °C for 3 h. After adding 3 volumes of 50 mM ABC buffer, the diluted samples were mixed with trypsin at a 1:40 (w/w) ratio and incubated at 37 °C for 18 h. The digested samples were acidified with 100% trifluoroacetic acid, followed by centrifugation at 14,000 g for 10 min. The supernatant was collected and desalted using a SepPak C18 column (Waters, Milford, MA). The desalted peptides were dried with a SpeedVac equipment, and the concentration was determined using the BCA assay.

LC-MS/MS measurement and data analysis. For proteome analysis, each dried peptide sample was suspended in 20 µL of 0.1% formic acid (FA) with 2% acetonitrile (ACN). A 2 µL aliquot of the sample (~500 ng) was analyzed in three technical replicates using an UltiMate 3000 UHPLC system (Thermo Fisher Scientific) coupled with a Thermo Fisher Scientific Q Exactive Orbitrap Mass Spectrometer. Peptides were separated on a 25-cm Thermo Acclaim PepMap column with a column heater set at 50 °C. The mobile phase consisted of buffer A (0.1% FA in deionized water) and buffer B (0.1% FA in 80% ACN), with each sample run using a 5%-30% ACN gradient over 180 minutes at a flow rate of 300 nL/min. Full MS scan spectra (m/z 400-2000) were acquired with the Orbitrap mass analyzer at a resolution of 70,000, using a maximum injection time of 50 ms and an AGC target of 3e6. The mass spectrometer operated in data-dependent acquisition mode, selecting up to 10 precursors for MS2 analysis with

a dynamic exclusion time of 30 seconds, normalized collision energy (NCE) of 27%, and an isolation window of 1.6 *m/z*.

To analyze the data, all 12 raw files were analyzed using Proteome Discoverer (ver. 2.4SP1) software against the SwissProt human protein database (20,213 entries, SwissProt TaxID=9606) with SequestHT search engines for the label-free quantification (LFQ). All MS spectra permitted a mass tolerance of 10 ppm for precursors, 0.02 Da for fragment masses, and a maximum of 2 missed cleavages for trypsin. Peptide identification stringency was set with a maximum FDR of 1% and a minimum peptide length of 6 residues. Oxidation (+15.995 Da) and phosphorylation (+79.966 Da) on Ser, Thr, and Tyr residues were included as dynamic modifications. The LFQ results at the protein level generated from Proteome Discoverer were further processed using Perseus software (version 2.0.6.0). Intensities of protein abundance were first log2 transformed, and accurately quantified proteome data were extracted from the identification of all triplicate replicates in at least one biological sample condition. A t-test was performed with a permutation-based FDR cutoff of 0.05 to identify significantly altered proteome datasets. The z-scores of these significantly changed proteins in abundance were used for hierarchical clustering analyses.

Protein-protein interactome by STRING analysis.

Protein-protein interactome analysis of differentially expressed proteins was conducted by using STRING database (version 11.5; https://string-db.org/). Interconnections of the mapped proteins were performed by MCL clustering with an inflation parameter of 2.0. The top-five key pathways enriched in each cluster with q-values less than 0.05 were further filtered out based on six commonly used functional frameworks, including Gene Ontology (https://amigo.geneontology.org/amigo/dd_browse), Reactome Pathways (https://reactome.org/), InterPro protein domains and features (https://www.ebi.ac.uk/interpro/), UniProt annotated keywords

(https://www.uniprot.org/), SMART protein domains (http://smart.embl-heidelberg.de/), and WikiPathways (https://www.wikipathways.org/).

2. Supporting data

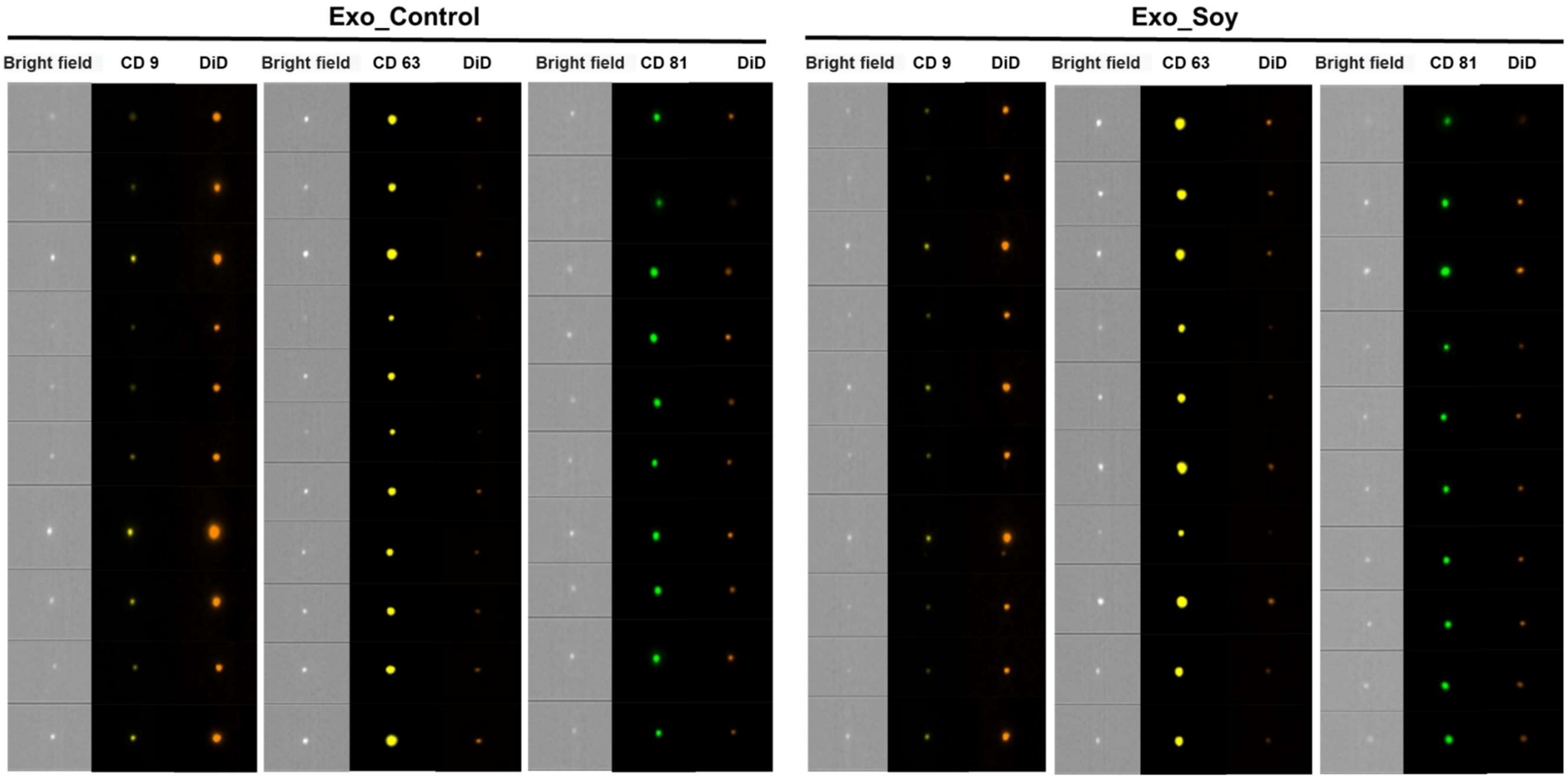

Figure S6 Imaging of exosomes. The Exo_Control and Exo_Soy exosome samples were labeled with the lipophilic tracer DiD to ensure the membrane integrity and counted in high-resolution fluorescence imaging flow analyzer (ImageStreamX Mark II, Amnis; NSTC Basic Research Core Facility, NYCU). Ten out of 10^4 counted exosomes/sample were shown. Along with DiD dye, exosomes were co-stained with antibodies corresponding to CD9, CD63, and CD81 to confirm the presence of these exosomal markers. Bright-field and fluorescent images are shown.

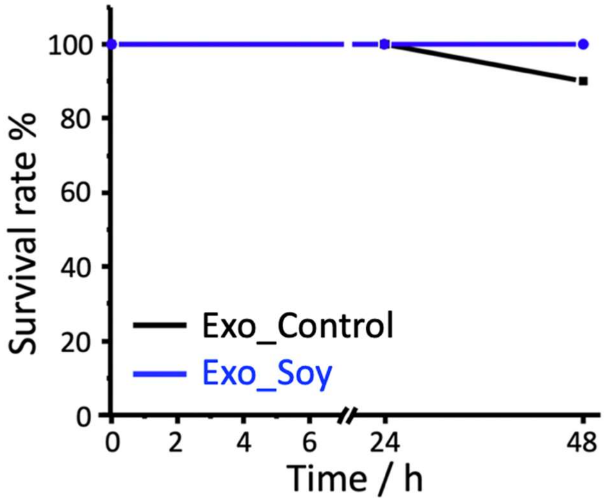

Figure S7 Toxicity assessment of WJ-MSC-secreted exosomes (collected from soy protein-coated and uncoated culture dishes) on zebrafish larvae survival. Zebrafish larvae were treated with either control exosomes (Exo_Control, black line) or soy protein-cultured exosomes (Exo_Soy, blue line), and survival rates were monitored over 48 hours at different time points. Sixty larvae were used in each group.

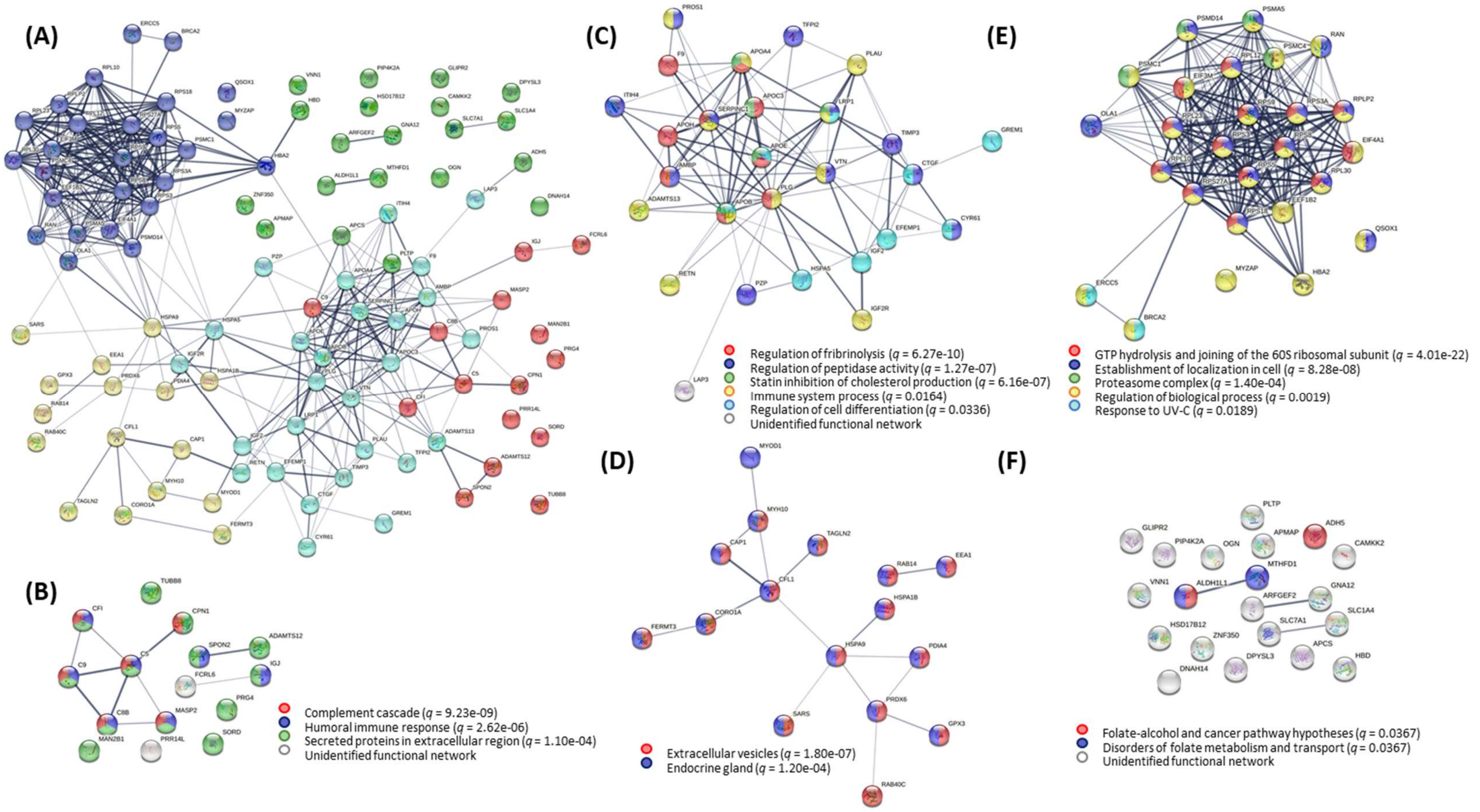

Figure S8 The highly up-regulated proteins identified in Exo_Soy. (A) Identified proteins can be categorized into 5 functional clusters (U1-U5). (B)-(F): Key pathways enriched in clusters of U1-U5 were analyzed based on STRING protein-protein database (https://string-db.org/).

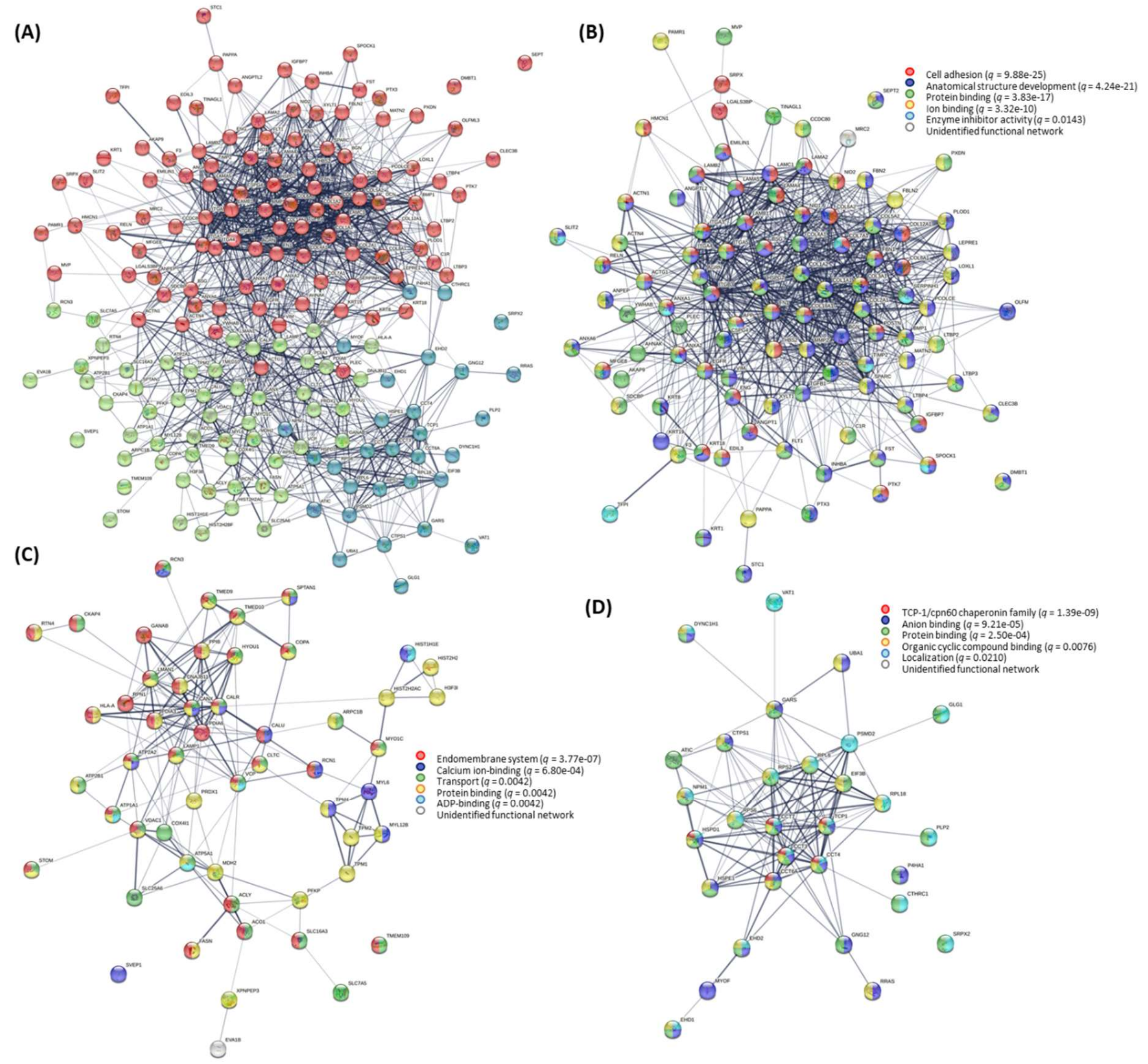

Figure S9 The highly down-regulated proteins identified in Exo_Soy. (A) Identified proteins can be categorized into 3 functional clusters (D1-D3). (B)-(D): Key pathways enriched in clusters of D1-D3 were analyzed based on STRING protein-protein database (https://string-db.org/).

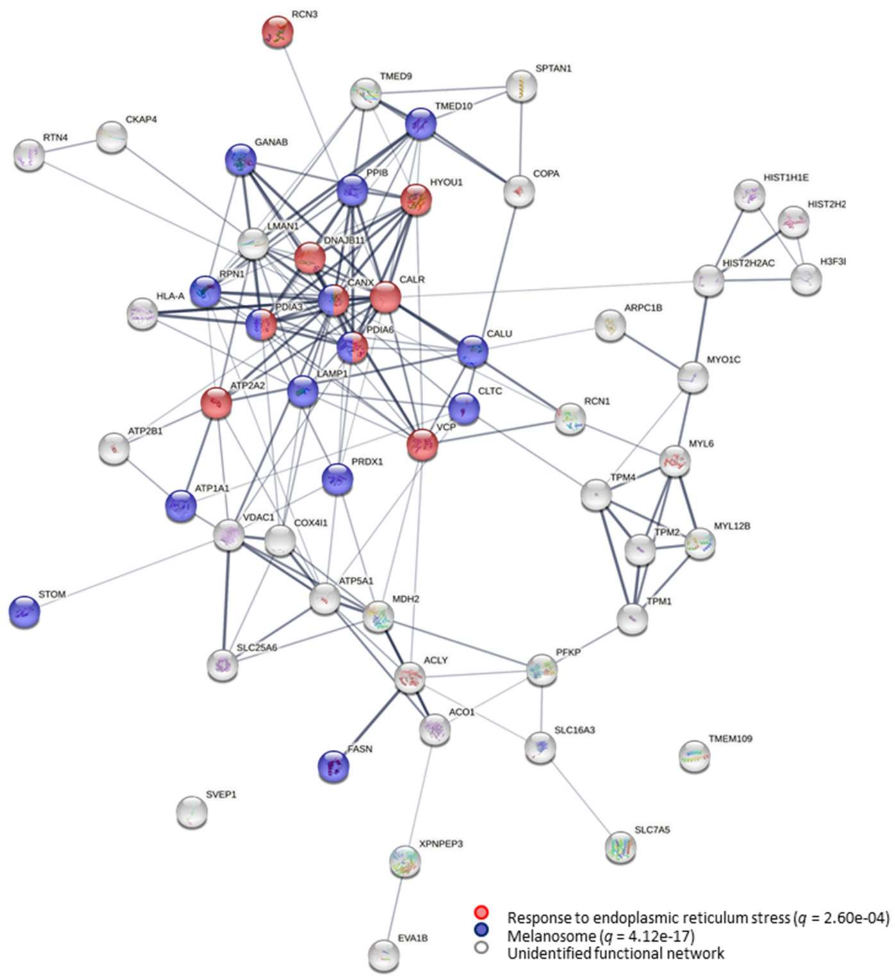

Figure S10 Down-regulated proteins in Exo_Soy are related to endomembrane and intracellular vesicle transportation systems (Cluster-D2). These proteins are involved in cell death triggered by ER stress (red-colored) and/or key components of melanosome which is a specialized autolysosome containing undegraded proteins and lipids in the human brain under oxidative stress.

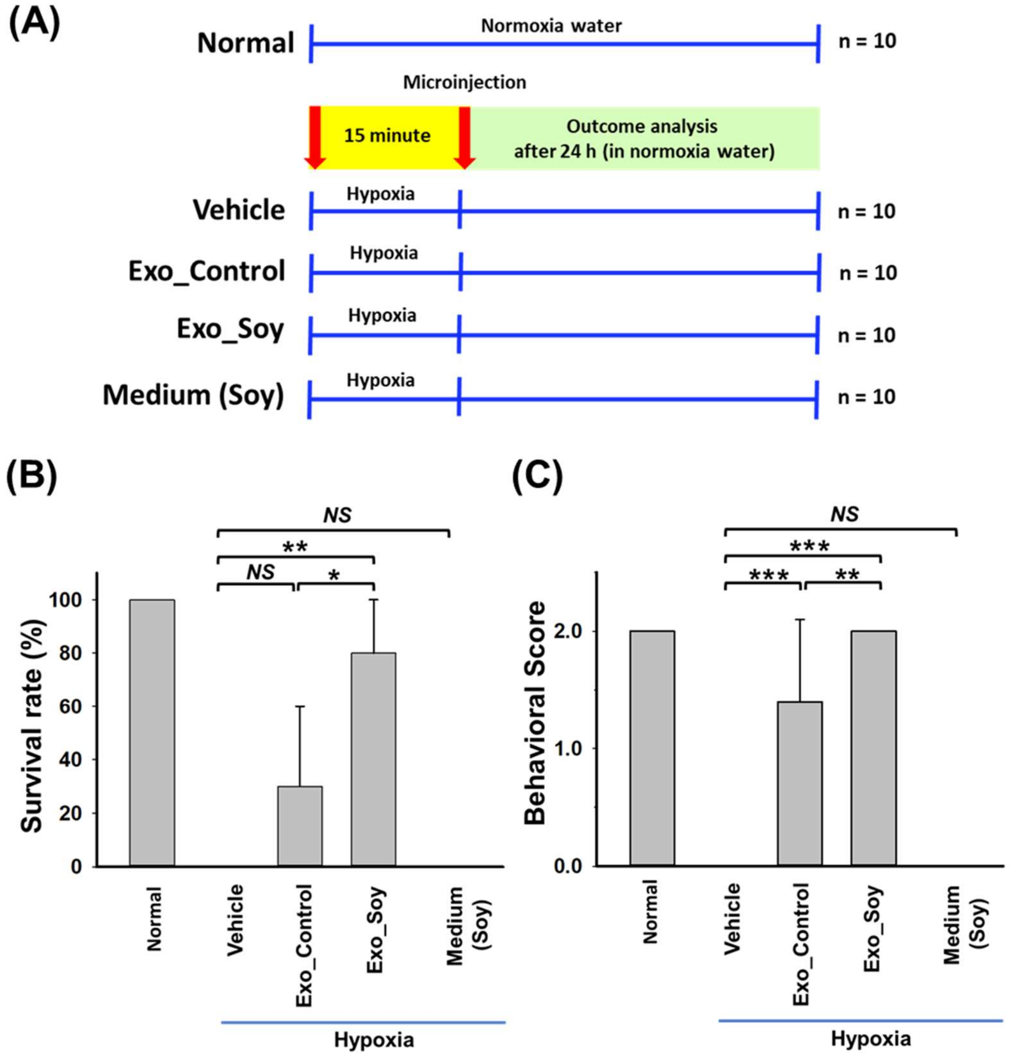

Figure S11 The culture medium (Soy) had no observable benefit in the assessment of larval survival and neurological function. (A) The protocol of indicated treatments in the hypoxia-induced injury zebrafish model. (B) Survival rate (%) of zebrafish larvae upon different indicated treatments post-hypoxia (24 h). (C) Behavioral assessment of neurological function evaluated post-hypoxia (24 h). Data are presented as mean ± SD (n = 10 larvae per group; *$p < 0.05$, **$p < 0.01$, and ***$p < 0.001$; NS indicates no significant difference ($p \geq 0.05$))

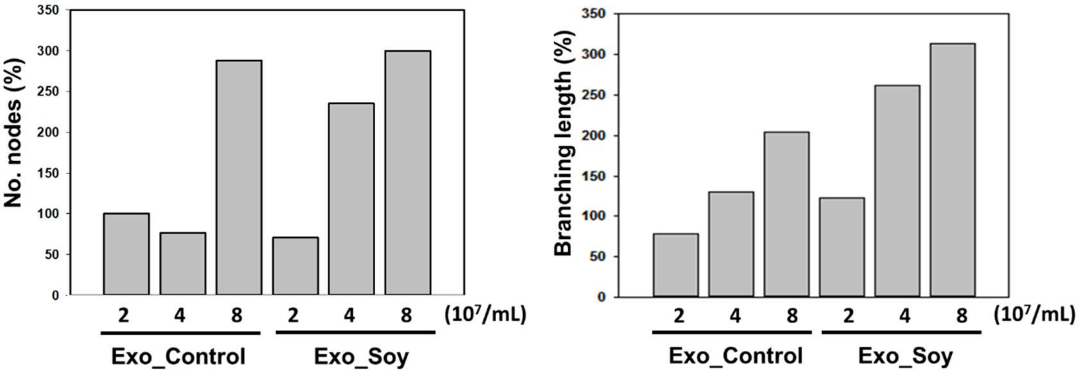

Figure S12 Dose-dependent effect of exosomes observed in a HUVEC tube formation assay. HUVECs (5× 10^3 cells/well) were cultured on Matrigel, followed by incubation with culture medium or exosome suspensions (Exo_Control or Exo_Soy) at indicated exosome concentration (2-8×10^7 particles/mL) for 24 hours. Images were captured using an inverted optical microscope and analyzed using the plugin "Angiogenesis Analyzer" in ImageJ (version 1.8.0). The percentages of the number of nodes and the branching length were quantified by dividing the data obtained in the exosome samples by the data in the control culture medium group. Increasing nodes and branching length indicated improved angiogenic potential.
